# Machine learning models of noninvasive tests to predict metabolic dysfunction–associated steatohepatitis and fibrosis stage

**DOI:** 10.1097/HC9.0000000000001064

**Published:** 2026-10-14

**Authors:** Rohit Loomba, Rebecca Taub, Dominic Labriola, Jan Priel, Krishna Padmanabhan, W. Ray Kim

**Affiliations:** 1 MASLD Research Center, Division of Gastroenterology and Hepatology, University of California at San Diego, La Jolla, California, USA; 2 Madrigal Pharmaceuticals, Inc., West Conshohocken, Pennsylvania, USA; 3 Cytel, Cambridge, Massachusetts, USA; 4 Mayo Clinic College of Medicine and Science, Phoenix, Arizona, USA

**Keywords:** biomarker, imaging, liver fibrosis, MASH, predictive learning model

## Abstract

**Background:**

Fibrosis stage in metabolic dysfunction–associated steatohepatitis (MASH) is currently diagnosed by liver biopsy in clinical trials. Using noninvasive tests, no consensus method exists for staging fibrosis or diagnosing MASH. We aimed to develop machine learning (ML) methods to predict fibrosis stage and MASH with F2/F3 fibrosis using noninvasive biomarkers.

**Methods:**

Data from 1970 biopsy-confirmed patients screened for phase 3 resmetirom trials (MAESTRO-NASH, MAESTRO-NAFLD-1) were retrospectively analyzed. Outcomes were fibrosis stage and MASH with F2/F3 fibrosis [NAFLD Activity Score (NAS) ≥4 with moderate to advanced noncirrhotic fibrosis], modeled from 37 clinical, laboratory, and imaging predictors. Random forest models were developed, including a full model using all 37 predictors and “Lean” models using subsets of predictors routinely available in clinical practice. These models were evaluated by 4-fold cross-validation using AUC.

**Results:**

For three-class fibrosis staging (F0/F1 vs. F2/F3 vs. F4), one-versus-rest cross-validation mean AUCs were 0.76, 0.75, and 0.94 for the full model and 0.83, 0.78, and 0.89 for the best-performing Lean model. Liver stiffness measures (LSM) and MASH composite algorithms, such as the Fibrosis-4 Index (FIB-4) and FibroScan-AST, were among the most important predictors. For MASH with F2/F3 fibrosis, the full and best-performing Lean models achieved AUCs of 0.75 and 0.79, respectively. Parsimonious top-k models retaining LSM preserved much of this performance.

**Conclusion:**

ML models using routinely available noninvasive biomarkers discriminated fibrosis stage and MASH with F2/F3 fibrosis with strong performance, supporting patient selection for clinical trials or practice. Lean predictor models that incorporated LSM outperformed models limited to blood-based biomarkers or a combination of FIB-4 and LSM. These proposed ML models need additional external and prospective validation before deployment for clinical use.

## Graphical abstract

**Figure:**
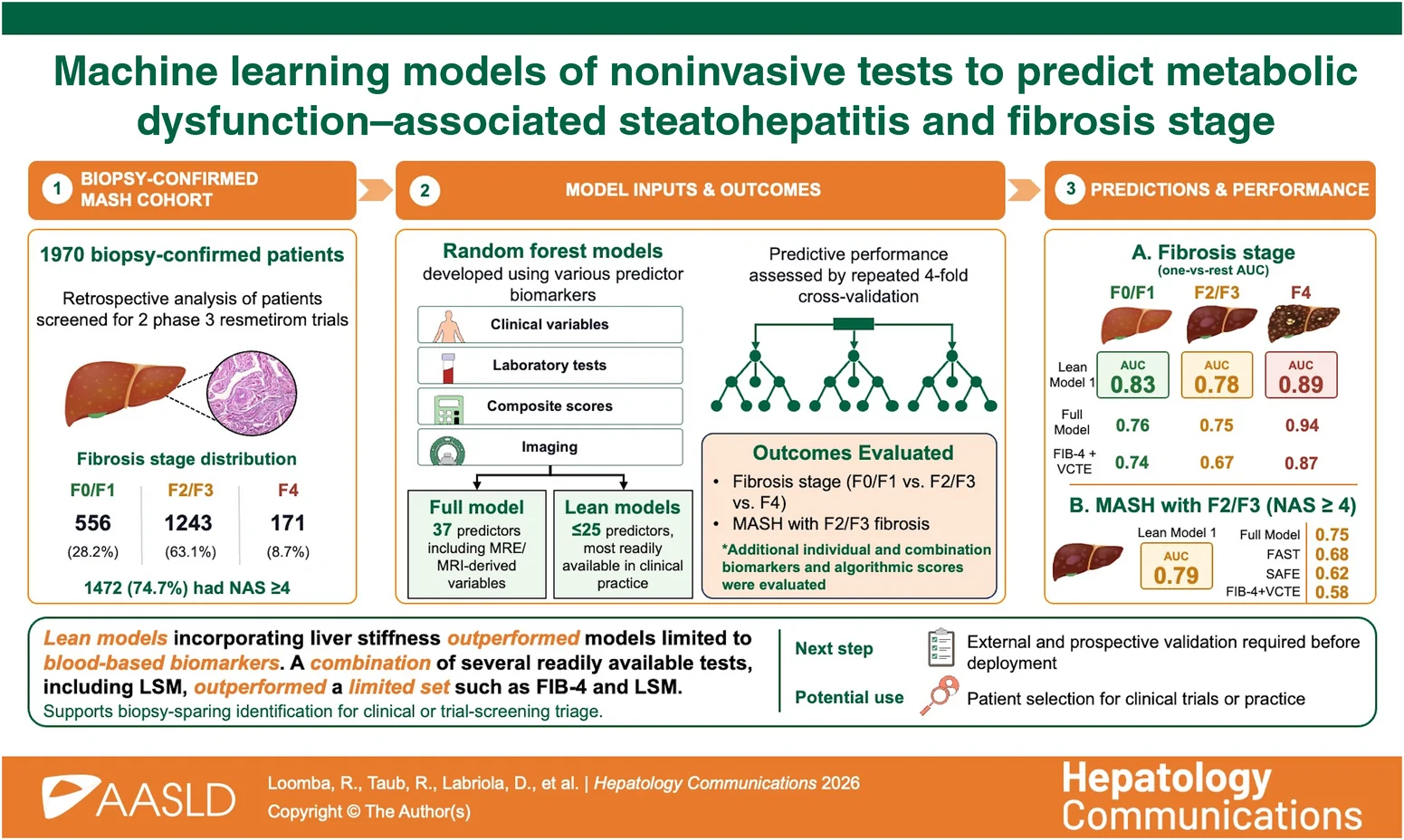

## INTRODUCTION

Non-alcoholic fatty liver disease (NAFLD), renamed as metabolic dysfunction–associated steatotic liver disease (MASLD), is the most prevalent cause of liver disease globally.^1^ MASLD is diagnosed by the presence of 5% or greater hepatic steatosis, either by imaging or biopsy, in the absence of any secondary causes of hepatic fat accumulation.^2^,^3^ MASLD is associated with metabolic dysregulation and is commonly identified in individuals with obesity, type 2 diabetes, hypertension, and dyslipidemia.^1^ The estimated global prevalence of MASLD is ~25%–30%.^4^

The more advanced form of MASLD, metabolic dysfunction–associated steatohepatitis (MASH), is a progressive liver disease characterized by 5% or greater hepatic steatosis with hepatic inflammation, hepatocellular ballooning, and fibrosis of varying severity [ie, none (F0), mild (F1), moderate (F2), advanced (F3), and cirrhosis (F4)].^5^ The estimated global prevalence of MASH is ~4%–6%, and the associated socioeconomic costs are high.^6^,^7^ Once MASH progresses to clinically meaningful fibrosis (stages F2 and F3), the risk of adverse clinical outcomes, including hepatocellular cancer, markedly increases, especially among patients with type 2 diabetes.^8^,^9^,^10^ A prospective study in adults with MASLD indicated that fibrosis stages F3 and F4 were associated with death due to liver disease, and other studies confirm risk in F2–F4 patients.^11^

Liver biopsy is a reliable approach for identifying the presence of steatohepatitis and fibrosis in patients with MASLD, but biopsy is limited by cost, infrequent real-world use, sampling error (with standard needle biopsy samples approximating only 1/50,000 of the mass of the liver), and procedure-related morbidity (and, very rarely, mortality).^3^ In clinical practice, liver biopsies are used to stage MASH infrequently. Therefore, noninvasive tests that can replace serial liver biopsies for identifying and following patients with MASH who are at risk of developing advanced fibrosis are urgently needed. These diagnostic tests have evolved significantly over the past several years to allow earlier patient identification.^12^,^13^,^14^ Resmetirom, a thyroid hormone receptor-beta agonist,^15^,^16^ and semaglutide,^17^ a glucagon-like peptide-1 agonist, are 2 drug therapies approved by the US Food and Drug Administration and European Medicines Agency that are now available for treatment of adults with noncirrhotic MASH and moderate to advanced liver fibrosis consistent with fibrosis stages F2 and F3, thereby supporting the diagnosis of MASH without a liver biopsy.

Fibrosis stage, the most important predictor of liver outcomes, may be predicted by a variety of different biomarkers and algorithms, including the Fibrosis-4 Index (FIB-4),^2^,^3^ Steatosis-Associated Fibrosis Estimator score (SAFE),^18^ FibroScan-AST score (FAST),^19^ and Enhanced Liver Fibrosis (ELF)^20^ panel and liver stiffness measures (LSM), including magnetic resonance elastography (MRE) and ultrasound-based transient elastography [eg, FibroScan vibration-controlled transient elastography (VCTE)]. Increased liver stiffness has been linked to liver outcomes and progression to cirrhosis.^21^,^22^,^23^ The relative importance and diagnostic accuracy of these biomarkers and imaging tools in predicting MASH and moderate to advanced fibrosis have been assessed in historic MASH cohorts and some prospective cohorts;^12^,^13^,^14^ however, these results have not been extensively validated in more recent well-characterized populations of patients with biopsy-proven MASH in clinical trials, which may incorporate a broader and more consistent array of biomarkers. Moreover, few studies have examined the ability of limited sets of biomarkers available in a real-world setting to accurately identify patients with MASH and moderate to advanced liver fibrosis.

The primary aim of the present study was to develop noninvasive prediction models to identify (a) fibrosis stage and (b) MASH [ie, NAFLD Activity Score (NAS) ≥4] with F2/F3 fibrosis based on analyses of a large cohort of biopsy-proven patients with F0 to F4 MASH enrolled in the phase 3 MAESTRO trials of resmetirom (NCT03900429^16^ and NCT04197479^15^). The purpose of noninvasive prediction models for fibrosis stage and MASH was to facilitate more accessible screening and diagnosis, thereby avoiding the need for liver biopsy in real-world and future clinical trial settings.

## METHODS

### Patient population

Data from 1970 patients screened for 2 phase 3 resmetirom trials, MAESTRO-NASH^16^ (ClinicalTrials.gov, NCT03900429) and MAESTRO-NAFLD-1^15^ (ClinicalTrials.gov, NCT04197479), who had baseline liver biopsy results, were included in this analysis. Screening criteria required adults (≥18 y) with 3 metabolic risk factors consistent with MASLD (diabetes/insulin resistance, hypertension, obesity/overweight, dyslipidemia), as defined by a modified International Diabetes Federation metabolic syndrome framework, and evidence of hepatic steatosis and fibrosis from recent assessments. Eligibility was supported by either a VCTE performed within 3 months before screening or a liver biopsy obtained within 6 months, and a screening MRI proton density fat fraction (MRI-PDFF) showing ≥8% liver fat content. Both trials were conducted in accordance with the principles of the Declaration of Helsinki and were approved by the institutional review board or ethics committee at participating sites, and patients provided written informed consent.^15^,^16^

In this work, MASH was defined as NAS ≥4. At-risk noncirrhotic fibrosis, per the US Prescribing Information for resmetirom, was defined as moderate to advanced liver fibrosis, consistent with stages F2 and F3. In this study, the F2/F3 fibrosis group combined F1B, F2, and F3 liver fibrosis.^3^ As prespecified in the MAESTRO-NASH statistical analysis plan, F1B, like F2, is visible on hematoxylin and eosin staining and therefore staged moderate compared with F1A/C (mild). F1B is distinguished from F2 only by location, not the amount of fibrosis.^5^ Pathologists have difficulty distinguishing between F1B and F2, given sampling variability and the small amount of portal fibrosis in many F2. Data from MAESTRO-NASH demonstrated that F1B met criteria for the “consistent with F2 stage” diagnosis based on several criteria: F1B and F2 were indistinguishable based on VCTE-assessed LSM (mean values of 11.3 kPa and 11.5 kPa, respectively) and MRE^16^ (mean values of 3.0 kPa and 3.1 kPa, respectively) and rate of progression to F3 stage, whereas F1 fibrosis (enrolled in the study as an exploratory population) showed LSM that were markedly lower (VCTE 10.4 kPa, MRE 2.6 kPa). Further validating the grouping for the current study, independent distribution profiles by fibrosis stages F0, F1, F1A, F1B, F2, F3, and F4 were generated for several biomarkers and confirmed that, although there was significant overlap of fibrosis stages, F1B had an indistinguishable profile from F2, whereas F1 and F0 showed a distribution shifted to a lower mean/median score.

Table 1 summarizes the baseline fibrosis stage and NAS distribution of the analysis population. The majority of patients had moderate to advanced fibrosis, and nearly three-quarters (74.7%) had a baseline NAS ≥4. A cohort of patients with MASH cirrhosis (F4 stage), most liver-biopsy confirmed, was also enrolled in MAESTRO-NAFLD-1, and additional patients with F4 were identified during screening for MAESTRO-NASH (screen failure cohort).

**TABLE 1:** Baseline fibrosis stage and NAFLD Activity Score distribution

	Baseline fibrosis stage					
	F0, N=243	F1, N=313	F2, N=592	F3, N=651	F4, N=171	Overall, N=1970
Baseline NAS, n (%)						
<4	165 (67.9%)	138 (44.1%)	68 (11.5%)	61 (9.4%)	37 (21.6%)	469 (23.8%)
≥4	77 (31.7%)	173 (55.3%)	524 (88.5%)	590 (90.6%)	108 (63.2%)	1472 (74.7%)
Missing	1 (0.4%)	2 (0.6%)	0 (0.0%)	0 (0.0%)	26 (15.2%)	29 (1.5%)

Abbreviation: NAS, NAFLD Activity Score.

Baseline fibrosis stage was from historic biopsies for the majority of the F4 population and based on eligibility read of a single central pathologist for the patients who screened failed MAESTRO-NASH. For eligible patients enrolled in MAESTRO-NASH, baseline fibrosis staging was determined by an independent read of a baseline liver biopsy, and if disparate, followed by a consensus review by 2 central pathologists. NAS was determined similarly for F4 and screen fail biopsies and for patients enrolled in MAESTRO-NASH using the eligibility read.

Our analysis focused on the classification of 2 separate outcomes at baseline:

- Outcome 1: Fibrosis stage (F0/F1 vs. F2/F3 vs. F4)—a classification exercise with 3 categories.
- Outcome 2: MASH with F2/F3 fibrosis, with MASH defined as a baseline NAS ≥4—a binary classification exercise.

Fibrosis stage was modeled as a three-level outcome, precluding a single performance metric. Instead, each class is evaluated against the others, yielding one-versus-rest AUCs and sensitivity and specificity estimates that characterize discrimination for each stage.

### Biomarker set

The set of predictor biomarkers was chosen, based on known risk factors, from the literature and from clinical laboratory assessment relevant for the diagnosis of fibrosis and MASH. For all biomarkers, we used baseline measurements before the first treatment of resmetirom or placebo.

Two different classes of models were investigated: full predictor models and “Lean” predictor models. The full predictor models utilized all 37 predictors, including MRE/MRI-derived variables (Table 2). The Lean predictor models utilized between 21 and 25 predictors, most of which are readily available in clinical practice. Lean predictor Model 1 included commonly available blood and imaging tests such as LSM (Table 2). Lean predictor Model 2 added the fibrosis-specific biomarker ELF and its components. Composite scores (eg, FAST) were included alongside their individual components because these algorithmic scores are available clinical tools that integrate information in ways familiar to practicing clinicians. Retaining them in the model preserves established heuristics and allows direct comparison of their relative importance against component biomarkers. Similar machine learning approaches have been applied to stage and grade MASH using clinical and biomarker predictors, through composite modeling of individual histological components from an observational cohort.^24^

**TABLE 2:** Variables included in each of the prediction models

			Prediction model (full, LM)			
Variable		Variable short name	Full	LM 1	LM 2	LM 3
1	Age	AGE	X	X	X	X
2	Sex	SEX	X	X	X	X
3	Race	RACE	X	X	X	X
4	Body mass index	BMI	X	X	X	X
5	Alcohol use	ALCOHOL	X	X	X	X
6	Tobacco use	TOBACCO	X	X	X	X
7	Hypertension	HTN	X			
8	Type 2 diabetes	T2D	X	X	X	X
9	VCTE	VCTE	X	X	X	
10	CAP score	CAP	X	X	X	
11	Enhanced Liver Fibrosis Test^a^	ELF	X		X	X
12	Hyaluronic acid	HA	X		X	X
13	Procollagen 3 N-terminal propeptide	P3NP	X		X	X
14	Tissue inhibitor of metalloproteinase 1	TIMP1	X		X	X
15	Sex hormone-binding globulin	SHBG	X			
16	M30 (CK18)	M30	X			
17	Adiponectin	APN	X			
18	Alkaline phosphatase	ALP	X	X	X	X
19	Aspartate aminotransferase	AST	X	X	X	X
20	Alanine aminotransferase	ALT	X	X	X	X
21	Gamma-glutamyl transferase	GGT	X	X	X	X
22	Thyroxine, free	FT4	X	X	X	X
23	MRI-PDFF	MRIPDFF	X			
24	MRE result, average	MRE	X			
25	MRI liver volume	LIVERVOL	X			
26	MRI spleen volume	SPLNVOL	X			
27	PRO-C3	PROC3	X			
28	FAST score (based on VCTE, CAP, AST)	FAST	X	X	X	
29	MAST score (based on MRE, MRI-PDFF, AST)	MAST	X			
30	FIB-4 score	FIB4	X	X	X	X
31	Reverse T3	RT3	X			
32	Free T3	T3FR	X	X	X	X
33	Free T3/Reverse T3 ratio	T3FR_RT3	X			
34	International normalized ratio	INR	X	X	X	X
35	Total bilirubin	BILI	X	X	X	X
36	Platelets	PLAT	X	X	X	X
37	Steatosis-associated fibrosis estimator score	SAFE	X	X	X	X

^a^ The Enhanced Liver Fibrosis panel consists of plasma levels of 3 matrix components (hyaluronic acid, tissue inhibitor of metalloproteinase 1, and amino-terminal of type III procollagen peptide).

Abbreviations: CAP, controlled attenuation parameter; CK18, cytokeratin 18; FAST, FibroScan-AST; FIB-4, Fibrosis-4; LM, Lean model; M30, caspase-cleaved cytokeratin; MAST, MRI-AST; MRE, magnetic resonance elastography; MRI-PDFF, MRI proton density fat fraction; PRO-C3, N-terminal type III collagen propeptide; T3, triiodothyronine; VCTE, vibration-controlled transient elastography.

### Model development and validation

We compared multiple supervised models, including regularized logistic regression, decision trees, random forest, boosted trees, Bayesian additive regression trees, k-nearest neighbors, discriminant analyses, and neural networks. Random forest^25^ achieved the highest mean AUCs with the most stable performance; Bayesian additive regression and boosted trees were similar but did not surpass random forest. Random forest models aggregate many decision trees to reduce variance and overfitting and are widely used in clinical prediction modeling for strong performance with minimal tuning.

To address class imbalance, we applied the Synthetic Minority Oversampling Technique (SMOTE)^26^ during training to synthesize minority-class examples via k-nearest neighbors; validation used un-augmented data. Predictors with >40% missingness were not imputed; analyses for these variables were restricted to observed values. For the remaining predictors, missForest^27^ imputed missing values by iteratively training a random forest until convergence. Model validation was performed on the original, unimputed data to limit bias.

Predictive performance was estimated with repeated K-fold cross-validation^28^ (*K*=4, *J*=100), yielding 400 test folds; we summarized results as mean (SD) AUC. Class thresholds were selected by minimizing Euclidean distance^29^ on the receiver operating characteristic (ROC) curve from the ideal (0,1) point. For fibrosis staging, we modeled the 3 classes in a one-versus-rest framework, estimating class-specific thresholds and assigning the class whose predicted probability exceeded its cut-off by the largest margin. To guard against information leakage to held-out data, predictor normalization, missForest imputation, and SMOTE oversampling were performed independently within each cross-validation training fold.

As sensitivity analyses, we also fit reduced random forest models restricted to the top 3, 5, and 10 predictors ranked by variable importance (mean decrease in Gini index) within the full and Lean Model 1 feature sets. These reduced models were trained and evaluated under the same repeated 4-fold cross-validation framework as the primary models. This process was not intended as a formal model-comparison or model-selection step, but rather as a descriptive illustration of the reduced model performance.

To complement global variable importance and enhance explainability of the random forest model, we used Accumulated Local Effects (ALE)^30^ plots to characterize how predictors shift class probabilities across the cohort, highlighting functional relationships and clinically relevant thresholds. For patient-level explanations, we computed Shapley values^31^ to quantify each predictor’s contribution to an individual classification, clarifying why a given fibrosis stage or MASH with F2/F3 fibrosis status was assigned.

This study takes into consideration the Transparent Reporting of a multivariable prediction model for Individual Prognosis Or Diagnosis (TRIPOD)^32^ guidance for prediction model development and internal validation. A complete TRIPOD checklist is provided in the Supplemental Materials, https://links.lww.com/HC9/C491.

## RESULTS

### Biomarker distribution

The prevalence and/or value of individual features/biomarkers according to fibrosis stage is shown in Supplemental Table S1, https://links.lww.com/HC9/C491. The mean age of the patients included was 56.4 years, with 46.4% male and 53.6% female; the majority (89.5%) were White. The mean body mass index (BMI) was 36.0 kg/m^2^; nearly 55% of patients had type 2 diabetes, and 49.0% had hypertension. Trends in the average values of the biomarkers by fibrosis stage can be seen in Supplemental Table S1, https://links.lww.com/HC9/C491. For example, between patients with F0 and F4 fibrosis, MRI-PDFF decreased from 19.1% to 10.7%, respectively, and MRE increased from 2.5 kPa to 5.5 kPa, respectively.

Baseline fibrosis stage was F0 in 12.3% of patients, F1 in 15.9%, F2 in 30.1%, F3 in 33.0%, and F4 in 8.7%. Note that 165 patients with F1B fibrosis were included among the F2 cohort (N=592), representing ~28% of this group. Among the 1970 screened patients with non-missing biopsy results, the number of patients with fibrosis stage F0/F1, F2/F3, and F4 was 556, 1243, and 171, respectively. The number of patients considered as having MASH (NAS ≥4) with F2/F3 fibrosis was 1114.

Since MRE/MRI was not performed on all screened patients, the variables had >40% missing value rates and were not imputed. Exclusion of these variables reduced the number of patients included in the full predictor models. MRE/MRI-PDFF were not included in the Lean predictor models; therefore, the missing MR values did not affect the performance of the Lean models. This likely affects direct performance comparisons between the full and Lean models. Any differences should be interpreted cautiously, as they may reflect not only feature availability but also differences in sample size and cohort composition resulting from missing MRI/MRE data. The resulting number of patients used for the development of full predictor models is provided in Supplemental Table S2, https://links.lww.com/HC9/C491. All 3 Lean models utilized data from the full set of 1970 patients.

### Predictive performance of the random forest model

The performance of all models was assessed on unseen data in repeated 4-fold cross-validation using the AUCs from a ROC curve. Mean (SD) one-versus-rest AUCs for fibrosis stage and for MASH with F2/F3 fibrosis are presented in Table 3. The full predictor model achieved strong discrimination for fibrosis stage, particularly for F4 versus rest [mean (SD) AUC of 0.94 (0.032)], but was less accurate for earlier stages [AUC (SD) of 0.76 (0.068) and 0.75 (0.055), respectively, for F0/1 vs. rest and F2/3 vs. rest] and for MASH with F2/F3 fibrosis [AUC (SD) of 0.75 (0.050)]. Among Lean models, Model 1 performed best, with AUCs of 0.83 (0.021), 0.78 (0.021), and 0.89 (0.026) for the respective fibrosis stage categories and 0.79 (0.020) for MASH with F2/F3 fibrosis. Lean Models 2 and 3 generally showed lower performance across outcomes, especially in predicting F2/F3 fibrosis, underscoring that Lean Model 1 offers the best balance of clinical practicality and predictive accuracy. Regarding models predicting MASH with F2/F3 fibrosis, the full model yielded a sensitivity of 71.1% (SD, 4.3%) and specificity of 65.1% (SD, 9.5%). This yields a positive predictive value (PPV) of 73.0% and a negative predictive value (NPV) of 63.8%. The sensitivity and specificity for the best-performing Lean model (Model 1) were 74.5% (SD, 2.71%) and 71.8% (SD, 3.16%), respectively. The PPV and NPV for this model were 77.4% and 68.6%, respectively (Supplemental Table S3A, https://links.lww.com/HC9/C491).

**TABLE 3:** Cross-validation AUC mean (SD) for fibrosis stage (one-vs.-rest) and MASH with F2/F3 fibrosis (yes/no) predictions

Model	Fibrosis stage (3-class prediction)			At-risk MASH
	F0/F1 vs. Rest	F2/3 vs. Rest	F4 vs. Rest	Yes vs. No
Full Model	0.76 (0.068)	0.75 (0.055)	0.94 (0.032)	0.75 (0.050)
Lean Model 1	0.83 (0.021)	0.78 (0.021)	0.89 (0.026)	0.79 (0.020)
Lean Model 2	0.71 (0.043)	0.69 (0.037)	0.91 (0.029)	0.66 (0.037)
Lean Model 3	0.68 (0.044)	0.64 (0.038)	0.85 (0.037)	0.62 (0.034)

Abbreviation: AUC, area under the receiver operating characteristic curve; MASH, metabolic dysfunction–associated steatohepatitis.

For models predicting fibrosis stages, the full model showed higher sensitivity for advanced fibrosis (F4; 82.3%) and an excellent NPV for F4 (98.1%), supporting its utility for ruling out cirrhosis. Lean Model 1 yielded consistently higher PPVs across classes compared with the full model (eg, 81.6% vs. 78.6%, respectively, for F2/F3) and higher sensitivity for early-stage disease (71.9% vs. 55.0%, respectively, for F0/F1). It also achieved higher specificity for F4 (91.6% vs. 86.6% for the full model). Notably, PPV for F4 was modest for both models (Lean, 41.5%; full, 37.3%), while NPVs were high for F4 with both models (Supplemental Table S3B, https://links.lww.com/HC9/C491).

### Evaluation of biomarker importance

Predictor importance was quantified using the mean decrease in Gini index from the trained random forest and displayed using variable importance plots. Figure 1A presents the results for the full model predicting fibrosis stage. For this model, the MRI-derived parameters MRE and MRI-PDFF were the 2 most important, followed by VCTE, FIB-4, free triiodothyronine/reverse triiodothyronine ratio, and platelets.

**FIGURE 1:**
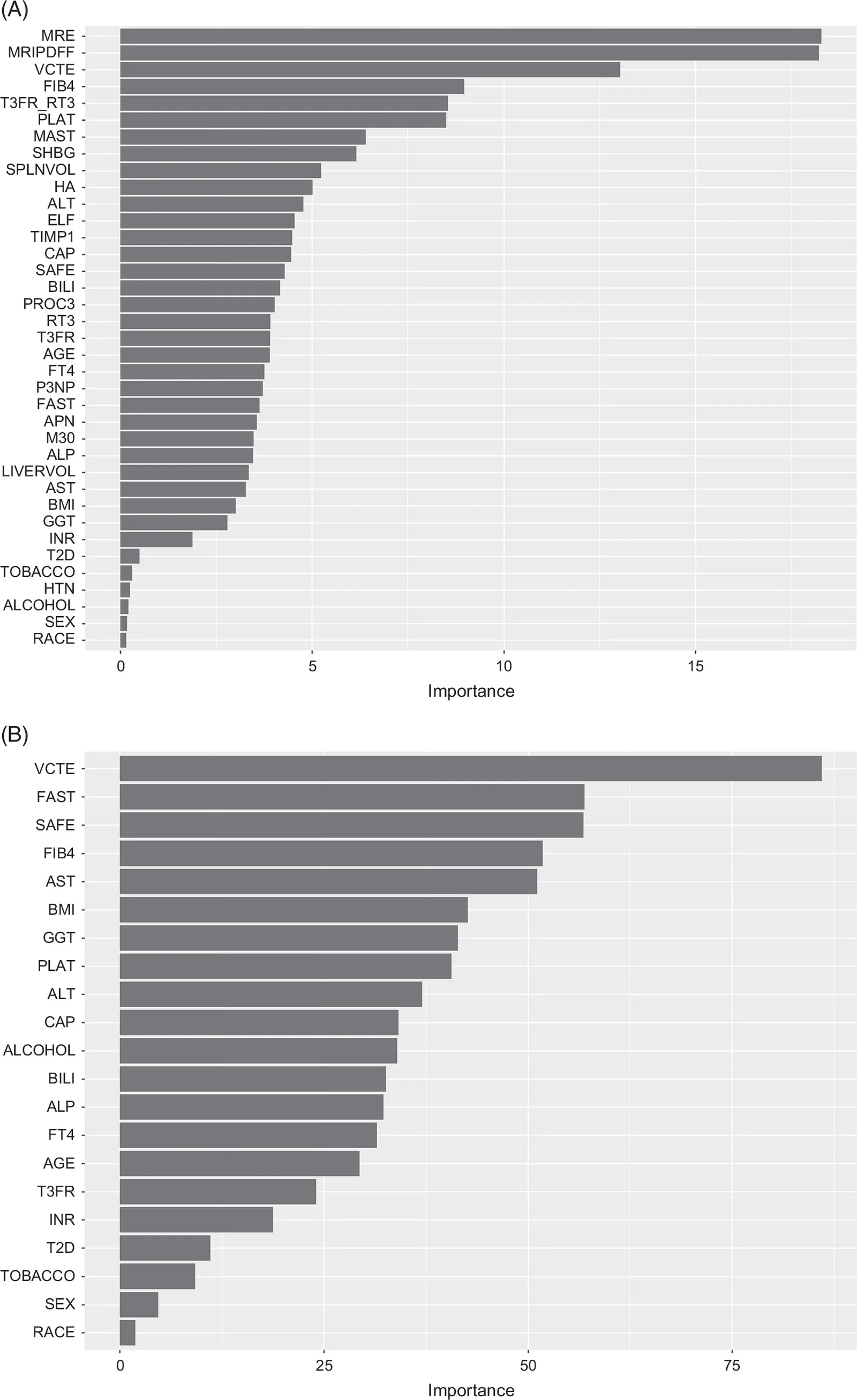
Random forest-based variable importance for fibrosis stage prediction: full model (A) and Lean Model 1 (B). Refer to Table 2 for expanded variables terminology.

Figure 1B shows random forest variable importance for Lean Model 1 in predicting fibrosis stage. VCTE, FAST, SAFE, and FIB-4 were found to be the most important predictors. Variable importance assessments for the fibrosis stage prediction for all assessed models can be found in Table 4. Supplemental Figures 1 and 2, https://links.lww.com/HC9/C491, show the variable importance plots for Lean Models 2 and 3 for fibrosis stage. The most important predictors for fibrosis stage included VCTE, SAFE, and FAST for Lean model 2 and SAFE, AST, and FIB-4 for Lean model 3.

**TABLE 4:** Variable importance in random forest models predicting baseline fibrosis stage

Full model	Lean model 1	Lean model 2	Lean model 3
Variable^a^	Variable^a^	Variable^a^	Variable^a^
MRE	VCTE	VCTE	SAFE
MRIPDFF	FAST	SAFE	AST
VCTE	SAFE	FAST	FIB4
FIB4	FIB4	AST	ELF
T3FR_RT3	AST	FIB4	P3NP
PLAT	BMI	P3NP	HA
MAST	GGT	ELF	GGT
SHBG	PLAT	HA	PLAT
SPLNVOL	ALT	GGT	ALT
HA	CAP	TIMP1	TIMP1
ALT	ALCOHOL	PLAT	BMI
ELF	BILI	BMI	ALCOHOL
TIMP1	ALP	ALCOHOL	ALP
CAP	FT4	ALT	BILI
SAFE	AGE	CAP	FT4
BILI	T3FR	BILI	AGE
PROC3	INR	ALP	T3FR
RT3	T2D	FT4	INR
T3FR	TOBACCO	AGE	T2D
AGE	SEX	T3FR	TOBACCO
FT4	RACE	INR	SEX
P3NP		T2D	RACE
FAST		TOBACCO	
APN		SEX	
M30		RACE	
ALP			
LIVERVOL			
AST			
BMI			
GGT			
INR			
T2D			
TOBACCO			
HTN			
ALCOHOL			
SEX			
RACE			

^a^ Refer to Table 2 for expanded variables terminology.

The most important predictors for MASH with F2/F3 fibrosis were similar to the fibrosis stage predictors in the full prediction model, whereas there were some differences versus Lean models. For the full model (Figure 2), MRI-PDFF, MRE, VCTE, and platelets were the most important predictors of MASH with F2/F3 fibrosis. For Lean Model 1, which also performed best for the prediction of MASH with F2/F3 fibrosis (Table 3), FAST, VCTE, alcohol consumption status, and AST were the most important predictive variables. Variable importance assessments for the prediction of MASH with F2/F3 fibrosis for all assessed models can be found in Supplemental Table S4, https://links.lww.com/HC9/C491.

**FIGURE 2:**
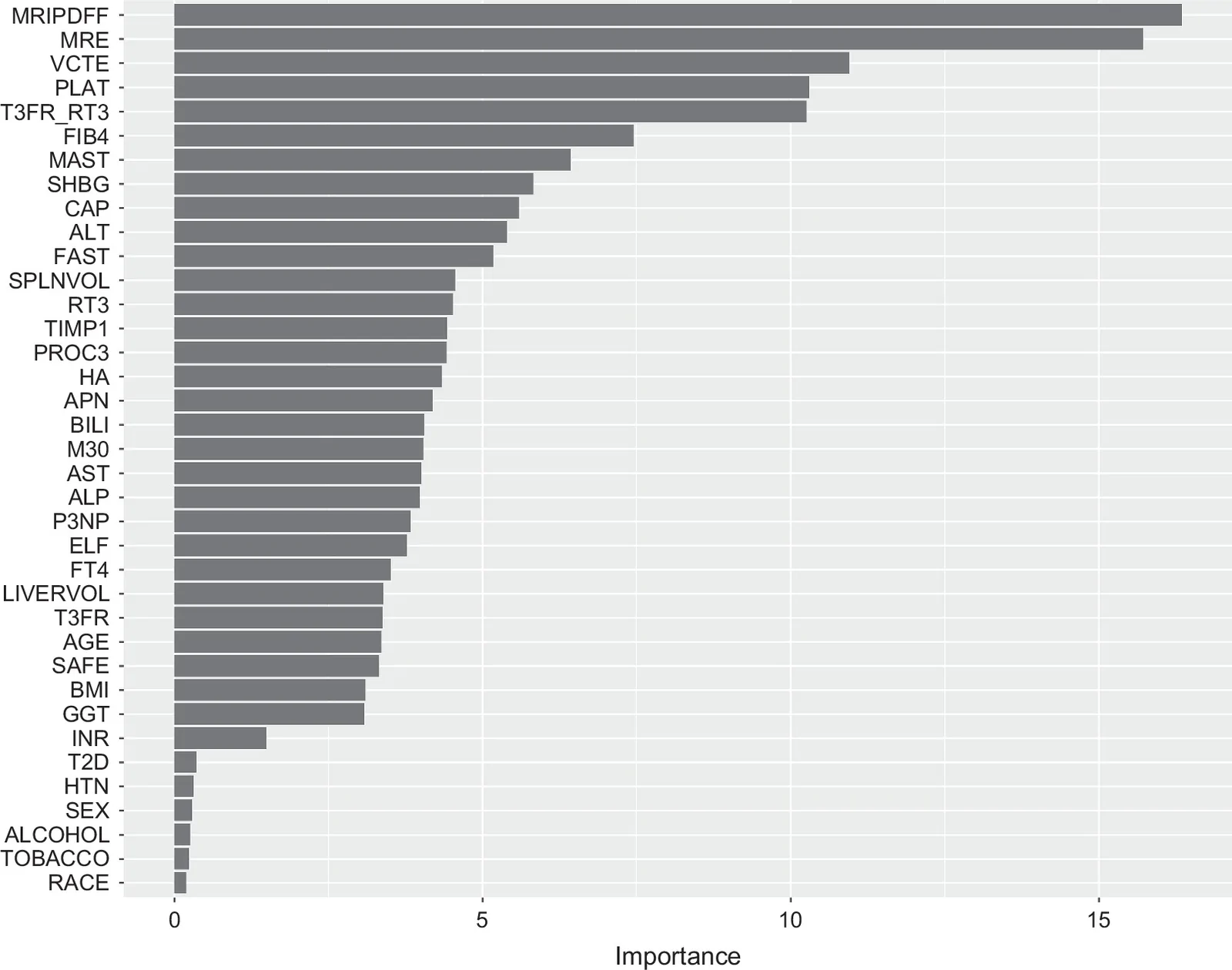
Full predictor model for MASH with F2/F3 fibrosis. Refer to Table 2 for expanded variables terminology. Abbreviation: MASH, metabolic dysfunction–associated steatohepatitis.

### Comparison with individual noninvasive tests and reduced combinations

To evaluate the random forest models in the context of established noninvasive tests, we evaluated individual biomarkers and algorithmic scores (MRE, VCTE, FIB-4, SAFE, FAST, and ELF) for fibrosis-stage classification within the same repeated 4-fold cross-validation framework, modeling each separately in a univariate multinomial logistic regression (Table 5). Full operating characteristics for the individual noninvasive tests, including sensitivity, specificity, PPV, and NPV for each fibrosis-stage class, are provided in Supplemental Table S5, https://links.lww.com/HC9/C491, and the corresponding values for identification of MASH with F2/F3 fibrosis are provided in Supplemental Table S6. Although sensitivity, specificity, PPV, and NPV were computed for every test, we focus on the AUC when comparing discrimination across models, as it is independent of any single decision threshold. PPV and NPV additionally depend on the chosen cut-off and an estimate of disease prevalence.

**TABLE 5:** Cross-validation AUC [mean (SD)] for individual noninvasive tests, a FIB-4 + VCTE combination, and the submitted random forest models, for fibrosis-stage (one-vs.-rest) and MASH with F2/F3 fibrosis

Identification of fibrosis stage			
Test or model	F0/F1 vs. Rest (AUC)	F2/F3 vs. Rest (AUC)	F4 vs. Rest (AUC)
Individual noninvasive tests			
MRE	0.82 (0.023)	0.72 (0.028)	0.89 (0.026)
VCTE	0.67 (0.023)	0.60 (0.025)	0.85 (0.027)
FIB-4	0.73 (0.021)	0.63 (0.024)	0.79 (0.034)
SAFE	0.73 (0.024)	0.63 (0.024)	0.75 (0.037)
FAST	0.72 (0.022)	0.65 (0.023)	0.61 (0.038)
ELF	0.64 (0.038)	0.56 (0.036)	0.76 (0.040)
Two-test combination			
FIB-4 + VCTE	0.74 (0.022)	0.67 (0.024)	0.87 (0.027)
Complete set of random forest models			
Full model	0.76 (0.068)	0.75 (0.055)	0.94 (0.032)
Lean Model 1	0.83 (0.021)	0.78 (0.021)	0.89 (0.026)
**Identification of MASH with F2/F3 fibrosis**			
**Test or Model**	**AUC**		
Individual noninvasive tests			
FAST	0.68 (0.022)		
SAFE	0.62 (0.022)		
MRE	0.61 (0.034)		
FIB-4	0.60 (0.024)		
MRI-PDFF	0.56 (0.026)		
ELF	0.51 (0.035)		
VCTE	0.45 (0.024)		
Two-test combination			
FIB-4 + VCTE	0.58 (0.023)		
Complete set of random forest models			
Full model	0.75 (0.050)		
Lean Model 1	0.79 (0.020)		

*Note:* Values are mean (SD) area under the receiver operating characteristic curve from repeated 4-fold cross-validation. Individual noninvasive tests and the FIB-4 + VCTE combination were each modeled by multinomial logistic regression using all patients with available data for the included test(s); missing values were not imputed. Random forest values are reproduced from Table 3 for reference.

Abbreviations: ELF, Enhanced Liver Fibrosis; FAST, FibroScan-AST; FIB-4, Fibrosis-4; MASH, metabolic dysfunction–associated steatohepatitis; MRE, magnetic resonance elastography; MRI-PDFF, MRI proton density fat fraction; SAFE, Steatosis-Associated Fibrosis Estimator score; VCTE, vibration-controlled transient elastography.

Across the 3 fibrosis-stage classes, no individual test matched the discrimination of Lean Model 1. MRE was the strongest single test, with one-versus-rest AUCs of 0.82, 0.72, and 0.89 for F0/F1, F2/F3, and F4, respectively, and was competitive for the F0/F1 and F4 classes; however, it did not exceed Lean Model 1 (0.83, 0.78, and 0.89) across classes and, unlike the Lean model, requires MR-based imaging. The blood-based tests (FIB-4, SAFE, ELF) and FAST showed weaker discrimination, particularly for the intermediate F2/F3 class (AUCs of 0.63, 0.63, 0.56, and 0.65, respectively).

Because current American Association for the Study of Liver Diseases (AASLD) guidance^3^ commonly recommends sequential risk stratification with FIB-4 followed by LSM, we also evaluated a two-variable FIB-4 + VCTE multinomial logistic regression model. This combination improved on either component alone (AUCs of 0.74, 0.67, and 0.87 for F0/F1, F2/F3, and F4, respectively) but remained below Lean Model 1 across all 3 classes (Table 5), indicating that the Lean Model 1 adds incremental value beyond a simple two-test strategy.

The blood-based biomarkers FIB-4 and ELF are also commonly used in combination within practices without access to LSM (eg, endocrinology) that are increasingly managing patients with MASH. A second combination of FIB-4 and ELF, 2 blood-based biomarkers in common use, was also evaluated. Evaluation of this pairing produced AUCs of 0.67, 0.62, and 0.82 for F0/F1, F2/F3, and F4, respectively, while SAFE + ELF produced AUCs for F1-F2/3-F4 of 0.65, 0.59, and 0.79, respectively. Across all 3 fibrosis classes, AUC values fell below both the FIB-4 + VCTE combination and Lean Model 1 (VCTE plus blood-based biomarkers) but were similar to Lean Model 3, which had no LSM (Table 3), further indicating that the multivariable Lean Model 1 and, specifically, LSM capture discriminatory information not recovered by either reduced two-test strategy.

For the binary endpoint of MASH with F2/F3 fibrosis, the advantage of the multivariable models was more pronounced (Table 5). No individual test approached the random forest models: FAST was the strongest single marker (AUC 0.68), followed by SAFE (0.62) and MRE (0.61), each well below the full and Lean Model 1 AUCs of 0.75 and 0.79. The FIB-4 + VCTE combination performed modestly (AUC 0.58) and did not improve meaningfully over the best individual tests, suggesting that this combination is not adequate for identifying MASH with F2/F3 fibrosis in this cohort.

### Performance of reduced top 3, 5, and 10 predictor random forest models

To assess whether smaller predictor sets, rather than the full set of biomarkers included in a Lean model, could retain performance, we refit random forest models restricted to the top 3, 5, and 10 predictors ranked by importance within the full and Lean Model 1 feature sets (Supplemental Tables S7 and S8, https://links.lww.com/HC9/C491).

For fibrosis staging, reduced predictor subsets retained much of the discrimination of the full model when MRI/MRE variables were included; for example, a top-5 subset of the full model (MRE, MRI-PDFF, VCTE, FIB-4, and the free T3/reverse T3 ratio) achieved AUCs of 0.82, 0.78, and 0.93. Within the Lean feature set, a 10-marker subset reached AUCs of 0.78, 0.73, and 0.88, modestly below Lean Model 1. For MASH with F2/F3 fibrosis, a 10-marker Lean subset achieved an AUC of 0.78, approaching the best-performing Lean Model 1 (0.79), while top-5 and top-10 subsets of the full model reached 0.78 and 0.81, respectively.

### Model explainability and clinical interpretation

We also used ALE plots to visualize the contributions of each of the predictor variables to the fibrosis stage classification. ALE plots quantify the main effect of a predictor on the outcome while isolating it from other variables, thereby providing a visualization that adjusts for correlations between predictors. The ALE plot for MRE, the most influential biomarker in the full model, and platelets, another strongly predictive marker, is shown in Figure 3A. At low MRE values (<2.5 kPa), predictions favored early-stage disease (F0/F1, red). Intermediate values (3–5 kPa) increased the probability of F2/F3 (green), while higher values (>5 kPa) sharply raised the probability of F4 (blue), consistent with literature thresholds. Reduced platelet count only showed prediction for F4, consistent with the reduction of platelets in patients with portal hypertension. ELF, a fibrosis progression marker with 3 individual components, was less important in discriminating between fibrosis stages; 1 of its components, hyaluronic acid, showed some ability to discriminate F4, while the other 2 components showed less impact. N-terminal type III collagen propeptide, a circulating biomarker with similarity to the ELF component amino-terminal of type III procollagen peptide, showed an increase in F4 probability (Supplemental Figure S3, https://links.lww.com/HC9/C491). The ALE plots for the 4 strongest predictors in the Lean model—VCTE, FIB-4, SAFE, and FAST—are depicted in Figure 3B. Higher VCTE and FIB-4 values shifted predictions toward advanced categories, particularly F4, while SAFE and FAST scores displayed discriminative patterns in the intermediate range, favoring F2/F3.

**FIGURE 3:**
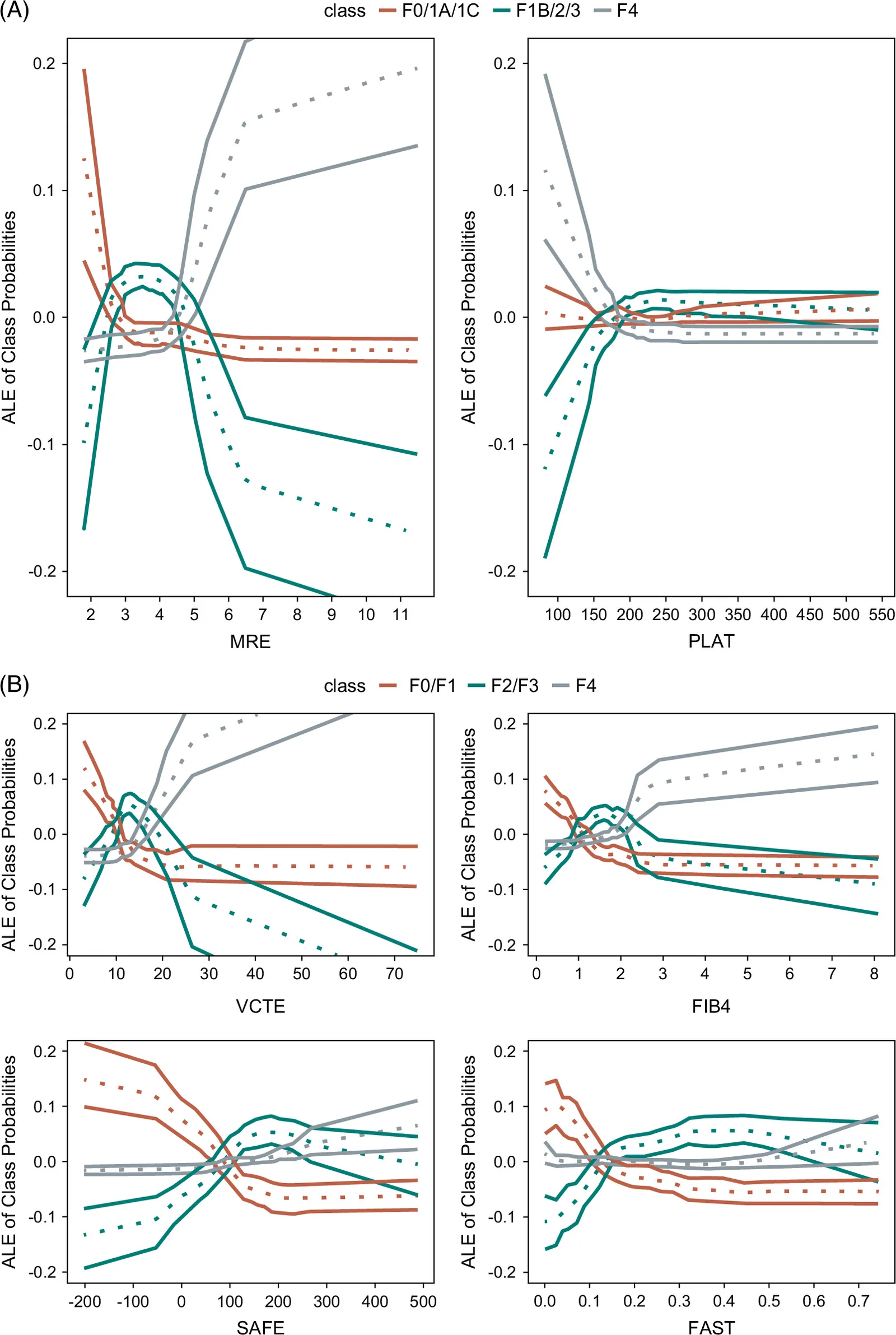
Accumulated local effects plots for key variables: MRE and platelets in the full model (A) and VCTE, FIB-4, SAFE, and FAST in Lean Model 1 (clockwise from top-left; B). Abbreviations: ALE, accumulated local effects; FAST, FibroScan-AST; FIB-4, Fibrosis-4; HA, hyaluronic acid; MRE, magnetic resonance elastography; P3NP, procollagen 3 N-terminal propeptide; PLAT, platelets; PRO-C3, N-terminal type III collagen propeptide; SAFE, Steatosis-Associated Fibrosis Estimator; VCTE, vibration-controlled transient elastography.

In addition, we used SHAP (SHapley Additive exPlanations) values to assess predictor contributions for individual patients. SHAP plots explain individual patient-level fibrosis stage predictions from the random forest model by quantifying how each clinical feature shifts the predicted risk or probability of a particular classification. Supplementary Figure 4 illustrates SHAP contributions for a correctly classified patient with fibrosis stage F3 (F2/F3 category). In this plot, bars extending right (in green) indicate positive contributions increasing class probability, while bars extending left (in red) decrease it. For this individual, the presence of alcohol history, an AST of 141 U/L, and a FAST of 0.7 had strongly positive contributions (green bars), supporting assignment to F2/F3 while also contributing against (red bars) classification to F0/F1. The VCTE of 21.8 kPa strongly decreased the assignment to F0/F1 while favoring the assignment to F4. Other contributions for F0/F1 and F4 were modest, and the combined effect of these led to the correct overall prediction. This patient-level perspective shows how the model integrates multiple biomarkers to reach a clinically consistent decision, complementing the cohort-level insights from ALE plots.

Supplemental Figure S5, https://links.lww.com/HC9/C491, illustrates how an isolated abnormal VCTE value can dominate a model’s risk estimate despite otherwise reassuring clinical data. Extreme obesity can further increase the risk of technically unreliable elastography measurements, warranting cautious interpretation of isolated VCTE outliers. The patient represented in this figure had a BMI of 58.4 kg/m^2^, was F0 stage on biopsy, and had no other markers suggestive of advanced fibrosis, yet a markedly elevated VCTE reading (~53 kPa) produced a large positive SHAP contribution toward the F4 class and strong negative contributions toward F0/F1 and F2/F3. This case underscores how elastography-related artifacts or technical errors can generate misleadingly high LSM and why clinical context and corroborating biomarkers are essential when interpreting predictions. In practice, discordant elastography results, especially at very high BMI, may warrant repeat measurement and/or confirmation with additional noninvasive tests rather than immediate escalation based on a single reading.

## DISCUSSION

In a large, biopsy-anchored cohort of 1970 patients spanning the MASLD/MASH spectrum, we developed and evaluated machine learning models that use only noninvasive data to predict both fibrosis stage and MASH with F2/F3 fibrosis. Two key findings emerged. First, a full model that included MRI/MRE measurements discriminated cirrhosis particularly well (F4-vs.-rest AUC of 0.94), highlighting the incremental value of MRE and MRI-PDFF when available. Second, and more clinically impactful, the best Lean model (Lean 1), incorporating tests routinely available in practice, delivered strong performance for both fibrosis staging (AUCs of 0.83, 0.78, and 0.89 for F0/F1, F2/F3, and F4, respectively) and identifying MASH with F2/F3 fibrosis (AUC 0.79; sensitivity 74.5%; specificity 71.8%). These results indicate that biopsy-sparing, clinic-ready risk stratification is feasible without advanced MR-based imaging, reserving MRE/MRI or liver biopsy for cases with diagnostic uncertainty or those requiring a cirrhosis rule-in.

These findings also emphasize the importance of including LSM. Lean Model 1, which incorporated VCTE with routine clinical and laboratory measures, outperformed blood-based biomarker combinations and Lean Model 3, which lacked LSM, indicating that elastography contributes discriminatory information not captured by circulating biomarkers or composite scores alone. Several predictors overlapped conceptually (eg, FIB-4 with AST/ALT, and FAST with VCTE/CAP/AST). Random forests are relatively robust to such redundancy, accommodating correlated and uninformative inputs without a loss of predictive performance.

Benchmarking against established tests reinforced the point that no individual test, including MRE, VCTE, FIB-4, SAFE, FAST, or ELF, matched the fibrosis-stage discrimination of Lean Model 1. An AASLD guideline-style FIB-4 + VCTE combination improved on either component alone but remained below Lean Model 1, particularly for the intermediate F2/F3 category. For MASH with F2/F3 fibrosis, the advantage of multivariable modeling was more pronounced: FAST was the strongest individual test (AUC 0.68), and FIB-4 + VCTE performed modestly (AUC 0.58), both below Lean Model 1 (AUC 0.79).

Reduced top-k random forest models for fibrosis staging and for MASH with F2/F3 fibrosis frequently approached the predictive accuracy of the complete model versions. These findings suggest that further exploration and validation of compact, routinely available predictor panels in future research could support simpler, more deployable risk-stratification tools.

Our results also speak to interpretability, which is often a barrier to clinical adoption of machine learning tools. ALE plots recapitulated clinically coherent relationships (eg, stepwise increases in predicted probability of advanced disease with rising LSM), while SHAP analyses provided patient-level explanations for individual classifications. ALE plots also provide a prediction for specific cut-off points to determine fibrosis stage; for example, VCTE recommendations of 8–10 kPa to <20 kPa to identify moderate to advanced noncirrhotic MASH aligns well with guideline documents^2^,^3^,^33^ and also demonstrates the uncertainty at the boundaries of these thresholds. Together, these methods show how the model “reasons,” supporting clinician trust and facilitating office-based decisions.

These findings have direct operational implications. Because the MAESTRO trials enriched the screened population for MASH using readily identifiable metabolic risk factors (diabetes, obesity, dyslipidemia, and hypertension) together with an LSM at the F1 to F2 boundary, and because these risk factors are themselves highly predictive of MASH rather than simple steatosis, comparable enrichment is achievable in routine care. The Lean model’s reliance on office-based elastography and routine laboratory tests, therefore, supports scalable, biopsy-sparing triage across primary care, endocrinology, hepatology, and gastroenterology. In practice, this enables a simple cascade: apply the routinely available Lean predictors first to stratify patients into low-risk, indeterminate-risk, and high-risk groups, reserve transient elastography and/or selective advanced imaging to confirm diagnosis, and reserve biopsy for discrepant findings or high-stakes decisions. An integrated, multi-marker approach also overcomes the limitation of single biomarkers such as platelet count or ELF, which are informative mainly at the advanced end of the fibrosis spectrum.

This work has several strengths. It combined two phase 3 biopsy-anchored populations with central pathologic review, yielding consistent phenotyping across the disease continuum and enabling direct comparison of full and Lean predictor sets. Model development followed best practices for imputation, class-imbalance handling, and repeated cross-validation (4-fold ×100), providing stable performance estimates. By evaluating both fibrosis staging and the composite MASH endpoint (NAS ≥4 with F2/F3 fibrosis), we targeted the populations most relevant to real-world triage and to selecting patients who may be candidates for approved therapies such as resmetirom or semaglutide (2.4 mg).

Several limitations should be considered. First, the MRI/MRE variables had >40% missingness, reducing the effective sample for the full model and likely attenuating its performance at earlier stages, whereas the Lean models used all 1970 participants, which may partly explain their robustness. Second, although repeated cross-validation provides robust internal validation, it does not ensure generalizability beyond the protocolized MAESTRO screening environment; external validation in independent cohorts is needed before clinical deployment, even as biopsy-anchored validation becomes more difficult with declining biopsy use. Third, the low prevalence of F4 fibrosis (8.7%) produced modest PPVs for cirrhosis (Lean, 41.5%; full, 37.3%) despite high NPVs; therefore, these models should be used as rule-out or triage tools rather than stand-alone rule-in tests for cirrhosis. Finally, the cohort was predominantly White (89.5%), limiting generalizability across demographic groups, and the Euclidean-optimized thresholds should be reconfigured to the intended use (eg, high-sensitivity rule-out vs. high-specificity rule-in).

At present, these models are not yet packaged as a deployable clinical tool, and their near-term value is likely in enriching enrollment for trials that use noninvasive criteria to identify MASH with F2/F3 fibrosis. Nonetheless, by integrating widely available noninvasive biomarkers with explainable methods (ALE and SHAP) and rigorous internal validation, this work demonstrates that interpretable, biopsy-sparing risk stratification is feasible. Lean models, including the reduced top-k random forest models, performed comparably to those requiring MR-based imaging. External and prospective validation are needed to confirm generalizability and clinical utility.

## Supplementary Material
