# Supplementary material for: Machine learning models of noninvasive tests to predict metabolic dysfunction–associated steatohepatitis and fibrosis stage
Source: Hepatol Commun. 2026 Oct 14;10(11):e01064. doi: 10.1097/HC9.0000000000001064 (PMC13668301; doi:10.1097/HC9.0000000000001064)
Supplement: Supplementary file 1 [file hc9-10-e01064-s001.pdf]

Supplementary Content

Machine Learning Models of Non-Invasive Tests to Predict Metabolic Dysfunction-Associated Steatohepatitis and Fibrosis Stage

Rohit Loomba, MD, MHSc[1], Rebecca Taub, MD[2], Dominic Labriola, PhD[2], Jan Priel, PhD[3], Krishna Padmanabhan, PhD[2], W. Ray Kim, MD[4]

[1]MASLD Research Center, Division of Gastroenterology and Hepatology, University of California at San Diego, La Jolla, CA, [2]Madrigal Pharmaceuticals, Inc., West Conshohocken, PA, [3]Cytel, Cambridge, MA, [4]Mayo Clinic College of Medicine and Science, Phoenix, AZ

Supplementary Table 1. Biomarker distribution in patient population

	Baseline Fibrosis Stage					
	F0<br>N = 243	F1<br>N = 313	F2<br>N = 592	F3<br>N = 651	F4<br>N = 171	Overall<br>N = 1970
Age, years	54.7 (12.4)	53.0 (12.9)	55.2 (11.8)	58.5 (9.9)	61.6 (8.6)	56.4 (11.5)
Sex, n (%)						
Female	126 (51.9%)	139 (44.4%)	320 (54.1%)	362 (55.6%)	109 (63.7%)	1056 (53.6%)
Male	117 (48.1%)	174 (55.6%)	272 (45.9%)	289 (44.4%)	62 (36.3%)	914 (46.4%)
Race, n (%)						
American Indian or Alaska Native	2 (0.8%)	1 (0.3%)	7 (1.2%)	4 (0.6%)	1 (0.6%)	15 (0.8%)
Asian	4 (1.6%)	6 (1.9%)	12 (2.0%)	20 (3.1%)	4 (2.3%)	46 (2.3%)
Black or African American	13 (5.3%)	12 (3.8%)	9 (1.5%)	15 (2.3%)	5 (2.9%)	54 (2.7%)
Native Hawaiian or Other Pacific Islander	0 (0.0%)	1 (0.3%)	2 (0.3%)	1 (0.2%)	0 (0.0%)	4 (0.2%)
White	217 (89.3%)	280 (89.5%)	537 (90.7%)	571 (87.7%)	158 (92.4%)	1763 (89.5%)
Other	6 (2.5%)	9 (2.9%)	17 (2.9%)	25 (3.8%)	2 (1.2%)	59 (3.0%)
Body Mass Index, kg/m^2	37.8 (8.1)	36.6 (6.7)	36.0 (6.8)	35.2 (6.6)	35.3 (6.6)	36.0 (6.9)
Alcohol Use, n (%)	35 (14.4%)	87 (27.8%)	313 (52.9%)	335 (51.5%)	65 (38.0%)	835 (42.4%)
Hypertension, n (%)						
Yes	33 (13.6%)	92 (29.4%)	333 (56.3%)	423 (65.0%)	84 (49.1%)	965 (49.0%)
No	19 (7.8%)	37 (11.8%)	156 (26.4%)	121 (18.6%)	24 (14.0%)	357 (18.1%)
Missing	191 (78.6%)	184 (58.8%)	103 (17.4%)	107 (16.4%)	63 (36.8%)	648 (32.9%)
Type 2 Diabetes, n (%)						
Present	84 (34.6%)	111 (35.5%)	339 (57.3%)	445 (68.4%)	97 (56.7%)	1076 (54.6%)
Absent	133 (54.7%)	161 (51.4%)	242 (40.9%)	195 (30.0%)	49 (28.7%)	780 (39.6%)
Missing	26 (10.7%)	41 (13.1%)	11 (1.9%)	11 (1.7%)	25 (14.6%)	114 (5.8%)
Fibroscan - VCTE, kPa	11.3 (6.3)	10.7 (5.9)	11.3 (5.6)	14.3 (6.5)	24.2 (13.3)	13.3 (7.9)
Fibroscan – CAP Score, dB/m	346.7 (35.6)	345.6 (38.3)	345.4 (38.2)	344.9 (39.6)	327.6 (52.8)	343.9 (40.1)
Enhanced Liver Fibrosis Test	9.3 (0.8)	9.4 (0.9)	9.5 (0.8)	10.0 (0.9)	10.6 (1.0)	9.8 (0.9)
PRO-C3, ng/mL	13.1 (4.7)	15.2 (6.2)	17.3 (7.1)	20.3 (11.3)	25.3 (15.2)	18.1 (9.7)
Tissue Inhibitor of Metalloproteinase 1, ng/mL	248.7 (54.9)	252.3 (60.1)	253.4 (64.8)	286.6 (76.8)	322.9 (104.1)	272.5 (76.2)
Alkaline Phosphatase, U/L	71.1 (21.7)	71.2 (27.4)	73.4 (25.0)	74.6 (27.1)	76.1 (31.8)	73.4 (26.4)
Aspartate Aminotransferase, U/L	24.0 (13.5)	29.5 (15.2)	37.6 (24.5)	41.8 (22.5)	40.0 (21.1)	36.3 (22.0)
Alanine Aminotransferase, U/L	35.4 (26.3)	47.1 (31.6)	54.2 (36.6)	54.4 (30.5)	43.9 (28.7)	49.9 (32.6)

Gamma Glutamyl Transferase, U/L	48.7 (50.2)	60.2 (71.6)	68.3 (74.9)	92.7 (111.8)	104.7 (115.5)	75.8 (91.6)
MRI-PDFF, %	19.1 (8.2)	19.5 (7.8)	18.0 (7.2)	16.4 (6.5)	10.7 (5.6)	17.2 (7.4)
MRE Result, kPa	2.5 (0.5)	2.7 (0.6)	3.1 (0.7)	4.0 (1.0)	5.5 (1.8)	3.5 (1.3)
MRI Liver Volume, mL	2326.9 (577.2)	2378.8 (547.9)	2365.9 (654.9)	2445.5 (664.0)	2324.0 (630.3)	2397.4 (650.3)
MRI Spleen Volume, mL	268.7 (119.7)	312.4 (112.1)	344.9 (162.1)	376.9 (189.9)	513.7 (252.4)	371.3 (188.0)
FAST Score	0.1 (0.1)	0.2 (0.1)	0.2 (0.1)	0.3 (0.2)	0.3 (0.2)	0.2 (0.2)
FIB-4 Score	0.9 (0.4)	1.0 (0.5)	1.2 (0.6)	1.6 (0.7)	2.3 (1.2)	1.4 (0.8)
Free T3/Reverse T3 Ratio	0.2 (0.1)	0.2 (0.1)	0.2 (0.1)	0.2 (0.1)	0.2 (0.1)	0.2 (0.1)
Steatosis-Associated Fibrosis Estimator Score	38.8 (89.9)	54.7 (85.9)	95.4 (86.5)	138.9 (87.2)	188.3 (92.7)	105.2 (97.4)

Abbreviations: CAP, controlled attenuation parameter; FAST, FibroScan-AST; FIB-4, Fibrosis-4; MRE, magnetic resonance elastography; MRI-PDFF, MRI proton density fat fraction; PRO-C3, N-terminal type III collagen propeptide; T3, triiodothyronine; VCTE, vibration-controlled transient elastography.

Supplementary Table 2. Patients by fibrosis stage and NAS included in the full predictor model

	Baseline Fibrosis Stage					
	**F0**<br>**N = 11**	**F1**<br>**N = 43**	**FF2**<br>**N = 278**	**F3**<br>**N = 312**	**F4**<br>**N = 87**	**Overall**<br>**N = 731**
Baseline NAS, n (%)						
<4	4 (36.4%)	4 (9.3%)	6 (2.2%)	5 (1.6%)	25 (28.7%)	44 (6.0%)
≥4	7 (63.6%)	39 (90.7%)	272 (97.8%)	307 (98.4%)	41 (47.1%)	666 (91.1%)
Missing	0 (0.0%)	0 (0.0%)	0 (0.0%)	0 (0.0%)	21 (24.1%)	21 (2.9%)

Abbreviation: NAS, NAFLD Activity Score.

Supplementary Table 3. Sensitivity, specificity, PPV, and NPV for full model and best lean model

A. Models predicting MASH with F2/F3 fibrosis

Model	Statistic	MASH with F2/F3 Fibrosis (Yes/No)
**Full Model**	Sensitivity	71.1% (4.3%)
	Specificity	65.1% (9.5%)
	PPV	73.0% (5.50%)
	NPV	63.8% (4.22%)
**Lean Model 1**	Sensitivity	74.5% (2.71%)
	Specificity	71.8% (3.16%)
	PPV	77.4% (1.93%)
	NPV	68.6% (2.29%)

Abbreviations: MASH, metabolic dysfunction-associated steatohepatitis; NPV, negative predictive value; PPV, positive predictive value.

B. Models predicting fibrosis stage

Model	Statistic	F0/F1 vs. Rest	F2/F3 vs. Rest	F4 vs. Rest
**Full Model**	Sensitivity	55.0% (14.79%)	63.3% (4.82%)	82.3% (11.19%)
	Specificity	79.3% (3.91%)	69.9% (9.12%)	86.6% (2.92%)
	PPV	50.4% (8.44%)	78.6% (5.20%)	37.3% (5.90%)
	NPV	82.2% (5.00%)	52.7% (4.21%)	98.1% (11.66%)
**Lean Model 1**	Sensitivity	71.9% (4.63%)	65.9% (3.45%)	62.2% (7.95%)
	Specificity	77.8% (2.56%)	74.5% (4.00%)	91.6% (1.78%)
	PPV	55.8% (2.97%)	81.6% (2.24%)	41.5% (5.21%)
	NPV	87.7% (1.74%)	56.2% (2.44%)	96.3% (0.74%)

Abbreviations: NPV, negative predictive value; PPV, positive predictive value.

Supplementary Table 4. Variable importance for random forest models predicting MASH with F2/F3 fibrosis

Full Model	Lean Model 1	Lean Model 2	Lean Model 3
**Variable[a]**	**Variable[a]**	**Variable[a]**	**Variable[a]**
MRIPDFF	FAST	FAST	AST
MRE	VCTE	P3NP	P3NP
VCTE	ALCOHOL	ALCOHOL	ALCOHOL
PLAT	AST	AST	ALT
T3FR_RT3	ALT	VCTE	TIMP1
FIB4	BMI	ALT	SAFE
MAST	SAFE	TIMP1	ELF
SHBG	PLAT	HA	HA
CAP	GGT	ELF	GGT
ALT	CAP	SAFE	BMI
FAST	FT4	GGT	PLAT
SPLNVOL	BILI	FIB4	FIB4
RT3	FIB4	PLAT	FT4
TIMP1	ALP	BMI	BILI
PROC3	AGE	CAP	ALP
HA	T3FR	FT4	AGE
APN	TOBACCO	BILI	T3FR
BILI	INR	AGE	TOBACCO
M30	T2D	ALP	INR
AST	SEX	T3FR	T2D
ALP	RACE	TOBACCO	SEX
P3NP		INR	RACE
ELF		T2D	
FT4		SEX	
LIVERVOL		RACE	
T3FR			
AGE			
SAFE			
BMI			
GGT			
INR			
T2D			
HTN			
SEX			
ALCOHOL			
TOBACCO			
RACE			

[a]Refer to Table 2 in main article for expanded variables terminology.

Supplementary Table 5. Cross-validation performance of individual non-invasive tests and a FIB-4 + VCTE combination, by fibrosis-stage class (one-vs.-rest)

Test or Model	Statistic	F0/F1 vs. Rest	F2/F3 vs. Rest	F4 vs. Rest
**Individual non-invasive tests**				
MRE	AUC	0.819 (0.023)	0.715 (0.028)	0.893 (0.026)
	Sensitivity	0.750 (0.052)	0.600 (0.037)	0.614 (0.089)
	Specificity	0.727 (0.031)	0.720 (0.046)	0.939 (0.015)
	PPV	0.520 (0.030)	0.787 (0.027)	0.494 (0.065)
	NPV	0.882 (0.020)	0.512 (0.025)	0.963 (0.008)
VCTE	AUC	0.673 (0.023)	0.596 (0.025)	0.853 (0.027)
	Sensitivity	0.653 (0.039)	0.486 (0.035)	0.518 (0.088)
	Specificity	0.623 (0.035)	0.678 (0.039)	0.918 (0.019)
	PPV	0.405 (0.021)	0.722 (0.022)	0.380 (0.052)
	NPV	0.821 (0.015)	0.434 (0.016)	0.953 (0.008)
FIB-4	AUC	0.734 (0.021)	0.628 (0.024)	0.790 (0.034)
	Sensitivity	0.691 (0.057)	0.515 (0.035)	0.373 (0.077)
	Specificity	0.642 (0.042)	0.671 (0.048)	0.939 (0.014)
	PPV	0.432 (0.021)	0.730 (0.023)	0.368 (0.053)
	NPV	0.843 (0.019)	0.446 (0.016)	0.941 (0.007)
SAFE	AUC	0.730 (0.024)	0.630 (0.024)	0.748 (0.037)
	Sensitivity	0.698 (0.043)	0.488 (0.037)	0.358 (0.075)
	Specificity	0.650 (0.024)	0.678 (0.041)	0.895 (0.025)
	PPV	0.438 (0.020)	0.723 (0.023)	0.250 (0.054)
	NPV	0.846 (0.018)	0.435 (0.018)	0.937 (0.007)
FAST	AUC	0.717 (0.022)	0.650 (0.023)	0.613 (0.038)
	Sensitivity	0.633 (0.040)	0.615 (0.029)	0.005 (0.014)
	Specificity	0.676 (0.027)	0.602 (0.037)	0.998 (0.005)
	PPV	0.434 (0.023)	0.727 (0.018)	0.348 (0.405)
	NPV	0.825 (0.015)	0.477 (0.021)	0.914 (0.001)

Test or Model	Statistic	F0/F1 vs. Rest	F2/F3 vs. Rest	F4 vs. Rest
ELF™	AUC	0.636 (0.038)	0.555 (0.036)	0.763 (0.040)
	Sensitivity	0.506 (0.072)	0.397 (0.046)	0.623 (0.098)
	Specificity	0.668 (0.040)	0.654 (0.065)	0.773 (0.030)
	PPV	0.374 (0.037)	0.665 (0.037)	0.207 (0.027)
	NPV	0.776 (0.025)	0.386 (0.021)	0.956 (0.010)
**Two-test combination**				
FIB-4 + VCTE	AUC	0.744 (0.022)	0.671 (0.024)	0.867 (0.027)
	Sensitivity	0.700 (0.040)	0.546 (0.029)	0.559 (0.073)
	Specificity	0.666 (0.024)	0.704 (0.035)	0.934 (0.013)
	PPV	0.451 (0.020)	0.760 (0.021)	0.448 (0.053)
	NPV	0.851 (0.017)	0.474 (0.018)	0.957 (0.007)

Values are mean (standard deviation [SD]) from repeated 4-fold cross-validation (K = 4, J = 100). Models are random forest classifiers restricted to the top 3, 5, and 10 predictors ranked by importance within the full and Lean Model 1 feature sets. Because the full and reduced full-feature models were restricted to patients with available MRI/MRE data, the values for these models are not directly comparable to those of the Lean models, which used all 1970 patients.

Abbreviations: ELF™, Enhanced Liver Fibrosis™; FAST, FibroScan-AST score; FIB-4, Fibrosis-4 Index; MRE, magnetic resonance elastography; NPV, negative predictive value; PPV, positive predictive value; SAFE, Steatosis-Associated Fibrosis Estimator score; VCTE, vibration-controlled transient elastography.

Supplementary Table 6. Cross-validation performance of individual non-invasive tests and a FIB-4 + VCTE combination for identification of MASH with F2/F3 fibrosis

Test or Model	Statistic	MASH with F2/F3 fibrosis
**Individual non-invasive tests**		
FAST	AUC	0.683 (0.022)
	Sensitivity	0.653 (0.029)
	Specificity	0.617 (0.032)
	PPV	0.689 (0.018)
	NPV	0.579 (0.021)
SAFE	AUC	0.622 (0.022)
	Sensitivity	0.622 (0.029)
	Specificity	0.578 (0.033)
	PPV	0.656 (0.018)
	NPV	0.541 (0.020)
MRE	AUC	0.614 (0.034)
	Sensitivity	0.625 (0.104)
	Specificity	0.561 (0.100)
	PPV	0.649 (0.026)
	NPV	0.547 (0.068)
FIB-4	AUC	0.597 (0.024)
	Sensitivity	0.593 (0.039)
	Specificity	0.566 (0.032)
	PPV	0.639 (0.018)
	NPV	0.519 (0.023)
MRI-PDFF	AUC	0.558 (0.026)
	Sensitivity	0.631 (0.051)
	Specificity	0.482 (0.030)
	PPV	0.612 (0.019)
	NPV	0.505 (0.035)

Test or Model	Statistic	MASH with F2/F3 fibrosis
ELF	AUC	0.505 (0.035)
	Sensitivity	0.522 (0.064)
	Specificity	0.499 (0.075)
	PPV	0.575 (0.046)
	NPV	0.446 (0.029)
VCTE	AUC	0.452 (0.024)
	Sensitivity	0.545 (0.126)
	Specificity	0.386 (0.132)
	PPV	0.530 (0.034)
	NPV	0.394 (0.023)
**Two-test combination**		
FIB-4 + VCTE	AUC	0.576 (0.023)
	Sensitivity	0.575 (0.038)
	Specificity	0.540 (0.038)
	PPV	0.619 (0.019)
	NPV	0.496 (0.022)

Values are mean (SD) from repeated 4-fold cross-validation (K = 4, J = 100). Models are random forest classifiers restricted to the top 3, 5, and 10 predictors ranked by importance within the full and Lean Model 1 feature sets. Because the full and reduced full-feature models were restricted to patients with available MRI/MRE data, the values for these models are not directly comparable to those of the Lean models, which used all 1970 patients.

Abbreviations: ELF, Enhanced Liver Fibrosis; FAST, FibroScan-AST score; FIB-4, Fibrosis-4 Index; MASH, metabolic dysfunction-associated steatohepatitis; MRE, magnetic resonance elastography; MRI, MRI proton density fat fraction; NPV, negative predictive value; PPV, positive predictive value; SAFE, Steatosis-Associated Fibrosis Estimator score; VCTE, vibration-controlled transient elastography.

Supplementary Table 7. Cross-validation performance of random forest models restricted to the top 3, 5, and 10 predictors, by fibrosis-stage class (one-vs.-rest)

Predictor set	Statistic	F0/F1 vs. Rest	F2/F3 vs. Rest	F4 vs. Rest
Full set, top 3: MRE, MRI-PDFF, VCTE	AUC	0.799 (0.025)	0.715 (0.028)	0.927 (0.021)
	Sensitivity	0.677 (0.059)	0.597 (0.039)	0.733 (0.082)
	Specificity	0.749 (0.033)	0.710 (0.048)	0.916 (0.017)
	PPV	0.514 (0.035)	0.780 (0.027)	0.456 (0.053)
	NPV	0.856 (0.022)	0.507 (0.025)	0.973 (0.008)
Full set, top 5: + FIB-4, free T3/reverse T3 ratio	AUC	0.824 (0.035)	0.782 (0.032)	0.934 (0.023)
	Sensitivity	0.675 (0.081)	0.694 (0.043)	0.803 (0.078)
	Specificity	0.827 (0.033)	0.744 (0.059)	0.909 (0.024)
	PPV	0.605 (0.046)	0.825 (0.032)	0.462 (0.065)
	NPV	0.868 (0.027)	0.587 (0.035)	0.980 (0.008)
Full set, top 10: + platelets, MAST, SHBG, MRI spleen volume, hyaluronic acid	AUC	0.806 (0.058)	0.801 (0.041)	0.934 (0.024)
	Sensitivity	0.636 (0.125)	0.685 (0.041)	0.827 (0.082)
	Specificity	0.842 (0.029)	0.772 (0.074)	0.873 (0.029)
	PPV	0.609 (0.066)	0.840 (0.043)	0.387 (0.057)
	NPV	0.857 (0.043)	0.588 (0.037)	0.982 (0.009)
Lean set, top 3: FAST, SAFE, VCTE	AUC	0.739 (0.023)	0.682 (0.022)	0.832 (0.032)
	Sensitivity	0.624 (0.048)	0.576 (0.035)	0.564 (0.085)
	Specificity	0.718 (0.031)	0.687 (0.040)	0.893 (0.019)
	PPV	0.465 (0.027)	0.760 (0.021)	0.336 (0.045)
	NPV	0.830 (0.017)	0.485 (0.020)	0.956 (0.008)
Lean set, top 5: + FIB-4, AST	AUC	0.757 (0.022)	0.700 (0.023)	0.861 (0.031)
	Sensitivity	0.653 (0.051)	0.590 (0.035)	0.589 (0.084)
	Specificity	0.722 (0.029)	0.700 (0.042)	0.906 (0.017)
	PPV	0.480 (0.024)	0.772 (0.022)	0.375 (0.046)
	NPV	0.842 (0.018)	0.499 (0.019)	0.959 (0.008)
	AUC	0.784 (0.022)	0.727 (0.024)	0.876 (0.030)

Predictor set	Statistic	F0/F1 vs. Rest	F2/F3 vs. Rest	F4 vs. Rest
Lean set, top 10: + BMI, GGT, platelets, ALT, CAP	Sensitivity	0.688 (0.048)	0.611 (0.032)	0.631 (0.082)
	Specificity	0.745 (0.027)	0.729 (0.040)	0.905 (0.016)
	PPV	0.514 (0.027)	0.795 (0.023)	0.389 (0.047)
	NPV	0.859 (0.017)	0.522 (0.022)	0.963 (0.008)

Values are mean (standard deviation [SD]) from repeated 4-fold cross-validation (K = 4, J = 100). Models are random forest classifiers restricted to the top 3, 5, and 10 predictors ranked by importance within the full and Lean Model 1 feature sets. Because the full and reduced full-feature models were restricted to patients with available MRI/MRE data, the values for these models are not directly comparable to those of the Lean models, which used all 1970 patients.

Abbreviations: BMI, body mass index; CAP, controlled attenuation parameter; FAST, FibroScan-AST score; FIB-4, Fibrosis-4 Index; GGT, gamma glutamyl transferase; MAST, MRI-AST; MRE, magnetic resonance elastography; MRI-PDFF, MRI proton density fat fraction; NPV, negative predictive value; PPV, positive predictive value; SAFE, Steatosis-Associated Fibrosis Estimator score; SHBG, sex hormone binding globulin; T3, triiodothyronine; VCTE, vibration-controlled transient elastography.

Supplementary Table 8. Cross-validation performance of random forest models restricted to the top 3, 5, and 10 predictors for identification of MASH with F2/F3 fibrosis

Predictor set	Statistic	MASH with F2/F3 fibrosis
Full set, top 3: MRI-PDFF, MRE, VCTE	AUC	0.693 (0.026)
	Sensitivity	0.667 (0.037)
	Specificity	0.628 (0.045)
	PPV	0.700 (0.025)
	NPV	0.594 (0.027)
Full set, top 5: + platelets, free T3/reverse T3 ratio	AUC	0.782 (0.032)
	Sensitivity	0.743 (0.040)
	Specificity	0.710 (0.056)
	PPV	0.770 (0.033)
	NPV	0.682 (0.034)
Full set, top 10: + FIB-4, MAST, SHBG, CAP, ALT	AUC	0.805 (0.028)
	Sensitivity	0.768 (0.039)
	Specificity	0.703 (0.055)
	PPV	0.772 (0.032)
	NPV	0.702 (0.035)
Lean set, top 3: FAST, VCTE, alcohol use	AUC	0.722 (0.020)
	Sensitivity	0.662 (0.030)
	Specificity	0.668 (0.033)
	PPV	0.721 (0.019)
	NPV	0.605 (0.021)
Lean set, top 5: + AST, ALT	AUC	0.733 (0.019)
	Sensitivity	0.673 (0.029)
	Specificity	0.688 (0.034)
	PPV	0.737 (0.020)
	NPV	0.620 (0.021)
Lean set, top 10: + BMI, SAFE, platelets, GGT, CAP	AUC	0.783 (0.019)

Predictor set	Statistic	MASH with F2/F3 fibrosis
	Sensitivity	0.723 (0.033)
	Specificity	0.710 (0.036)
	PPV	0.764 (0.020)
	NPV	0.665 (0.024)

Values are mean (SD) from repeated 4-fold cross-validation (K = 4, J = 100). Models are random forest classifiers restricted to the top 3, 5, and 10 predictors ranked by importance within the full and Lean Model 1 feature sets. Because the full and reduced full-feature models were restricted to patients with available MRI/MRE data, the values for these models are not directly comparable to those of the Lean models, which used all 1970 patients.

Abbreviations: BMI, body mass index; CAP, controlled attenuation parameter; FAST, FibroScan-AST score; FIB-4, Fibrosis-4 Index; GGT, gamma glutamyl transferase; MASH, metabolic dysfunction-associated steatohepatitis; MAST, MRI-AST; MRE, magnetic resonance elastography; MRI-PDFF, MRI proton density fat fraction; NPV, negative predictive value; PPV, positive predictive value; SAFE, Steatosis-Associated Fibrosis Estimator score; SHBG, sex hormone binding globulin; T3, triiodothyronine; VCTE, vibration-controlled transient elastography.

Supplementary Figure 1. Random forest–based variable importance for fibrosis stage prediction: Lean Model 2

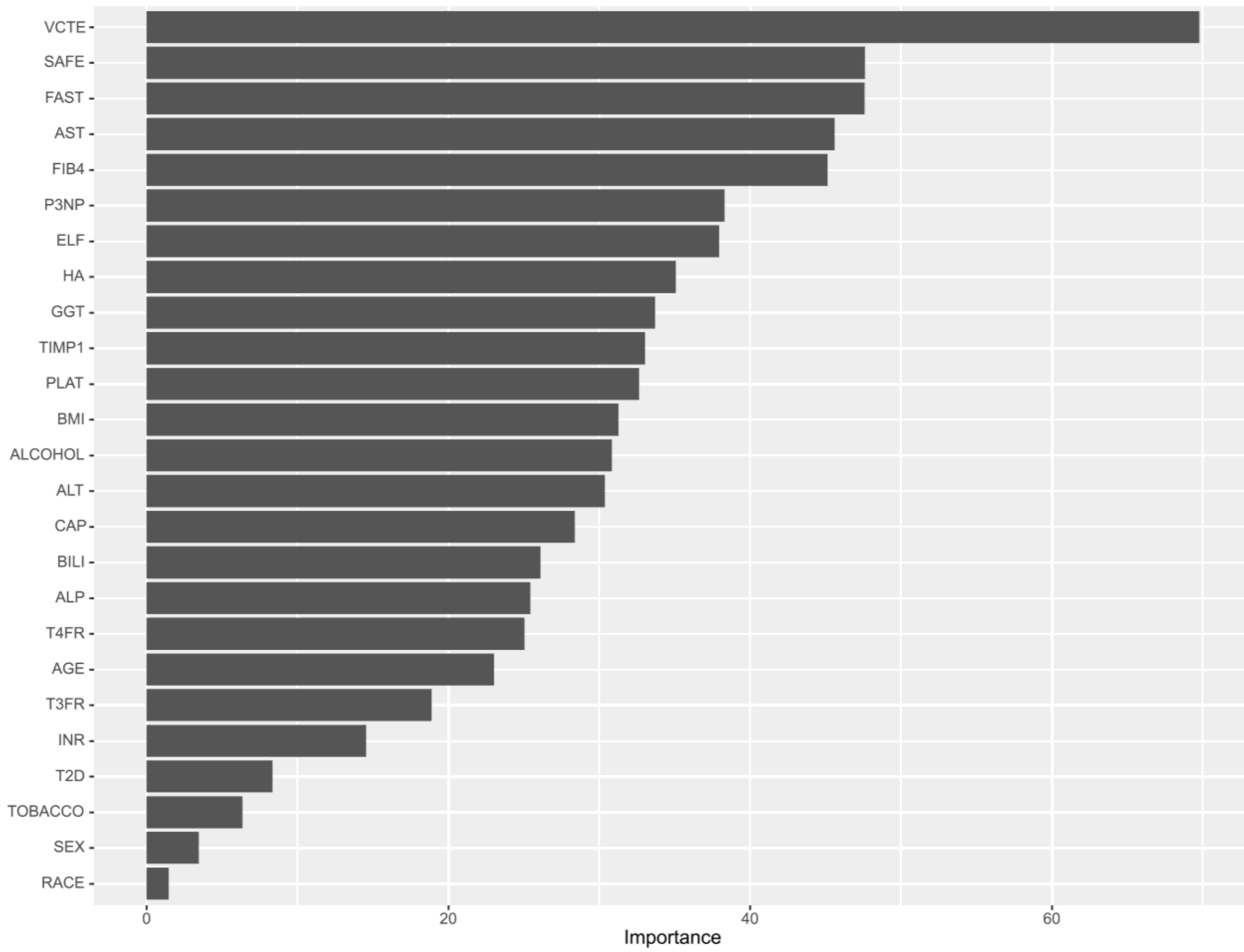

Supplementary Figure 2. Random forest–based variable importance for fibrosis stage prediction: Lean Model 3

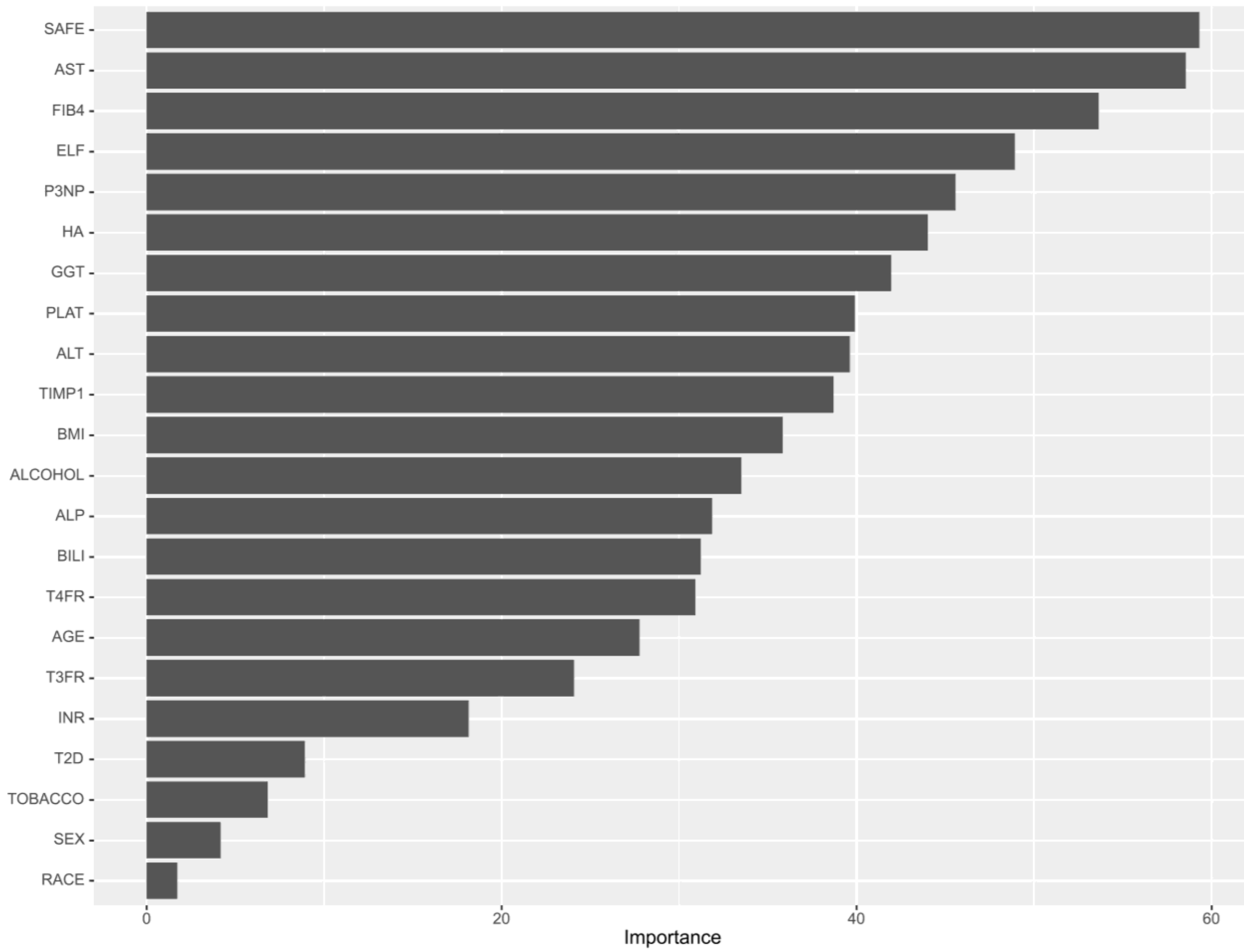

Supplementary Figure 3. ELF, ELF components, and PRO-C3 in full random forest model

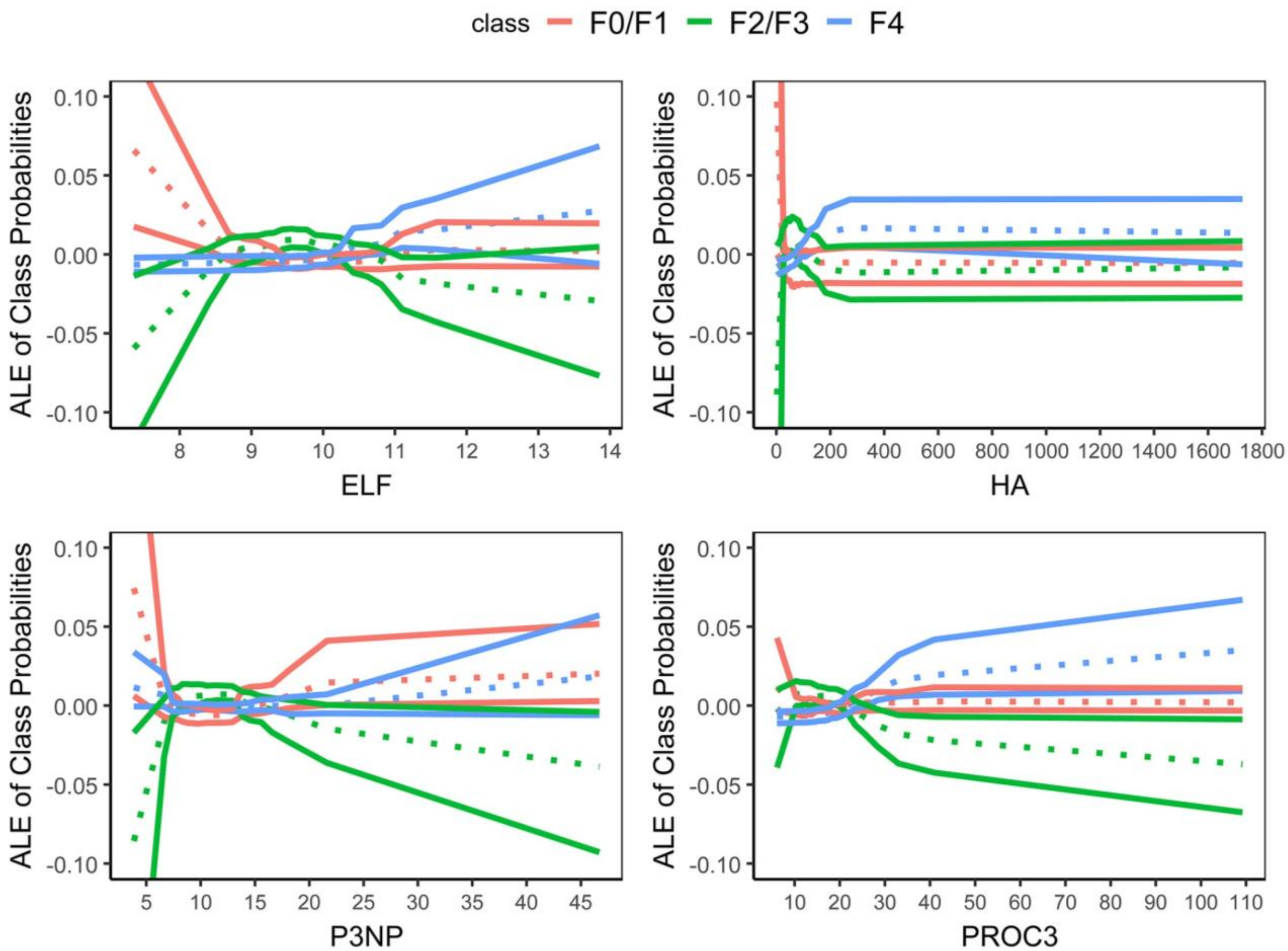

Abbreviations: ALE, Accumulated Local Effects; ELF, Enhanced Liver Fibrosis; HA, hyaluronic acid; P3NP, procollagen 3 N-terminal propeptide; PRO-C3, N-terminal type III collagen propeptide.

Supplementary Figure 4. Shapley values explaining feature contributions in a correctly classified F3 patient (Lean Model 1)[a]

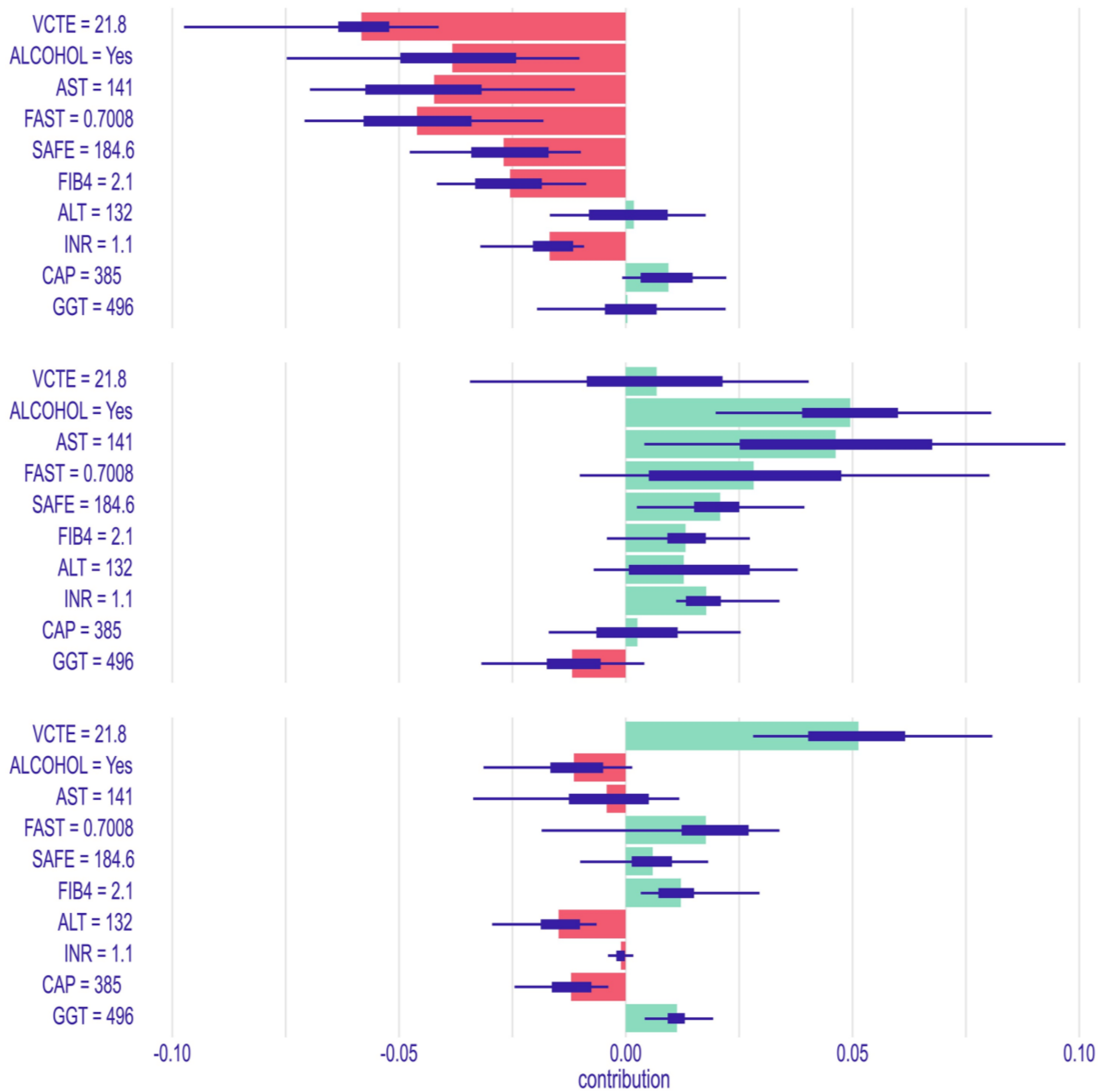

[a]Refer to Table 2 in main article for expanded variables terminology.

Supplementary Figure 5. Shapley values explaining feature contributions in an incorrectly classified F0 patient (Lean Model 1)[a]

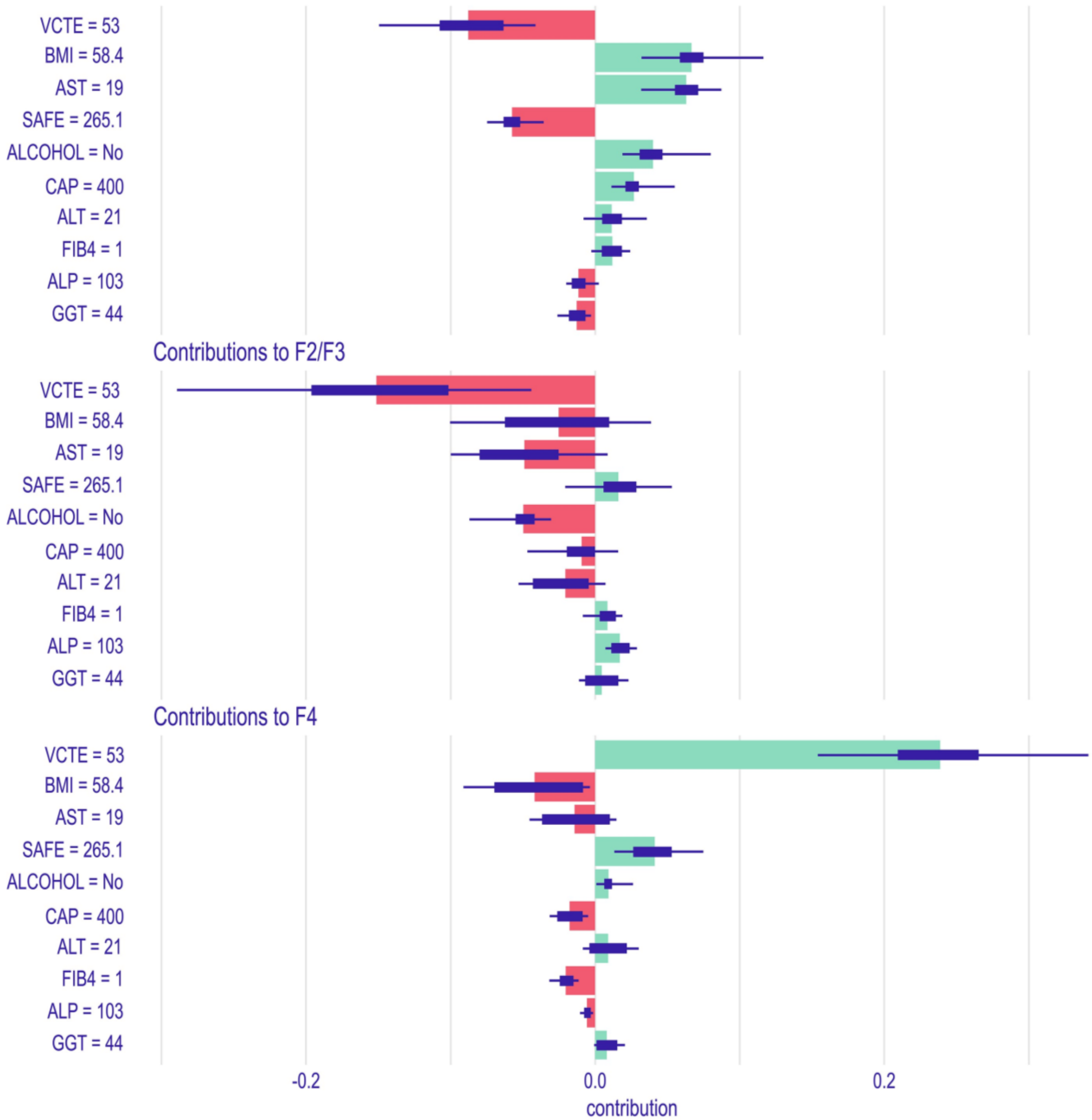

[a]Refer to Table 2 in main article for expanded variables terminology.

Section/Topic	Item	Development / evaluation[1]	Checklist item	Reported on page
**TITLE**				
Title	1	D;E	Identify the study as developing or evaluating the performance of a multivariable prediction model, the target population, and the outcome to be predicted	1
**ABSTRACT**				
Abstract	2	D;E	See TRIPOD+AI for Abstracts checklist	3
**INTRODUCTION**				
Background	3a	D;E	Explain the healthcare context (including whether diagnostic or prognostic) and rationale for developing or evaluating the prediction model, including references to existing models	4-5
	3b	D;E	Describe the target population and the intended purpose of the prediction model in the context of the care pathway, including its intended users (e.g., healthcare professionals, patients, public)	5
	3c	D;E	Describe any known health inequalities between sociodemographic groups	-
Objectives	4	D;E	Specify the study objectives, including whether the study describes the development or validation of a prediction model (or both)	5
**METHODS**				
Data	5a	D;E	Describe the sources of data separately for the development and evaluation datasets (e.g., randomised trial, cohort, routine care or registry data), the rationale for using these data, and representativeness of the data	5,15-16
	5b	D;E	Specify the dates of the collected participant data, including start and end of participant accrual; and, if applicable, end of follow-up	-
Participants	6a	D;E	Specify key elements of the study setting (e.g., primary care, secondary care, general population) including the number and location of centres	5
	6b	D;E	Describe the eligibility criteria for study participants	5-6
	6c	D;E	Give details of any treatments received, and how they were handled during model development or evaluation, if relevant	5
Data preparation	7	D;E	Describe any data pre-processing and quality checking, including whether this was similar across relevant sociodemographic groups	-
Outcome	8a	D;E	Clearly define the outcome that is being predicted and the time horizon, including how and when assessed, the rationale for choosing this outcome, and whether the method of outcome assessment is consistent across sociodemographic groups	4,5,6
	8b	D;E	If outcome assessment requires subjective interpretation, describe the qualifications and demographic characteristics of the outcome assessors	-
	8c	D;E	Report any actions to blind assessment of the outcome to be predicted	-
Predictors	9a	D	Describe the choice of initial predictors (e.g., literature, previous models, all available predictors) and any pre-selection of predictors before model building	7-8
	9b	D;E	Clearly define all predictors, including how and when they were measured (and any actions to blind assessment of predictors for the outcome and other predictors)	7,21
	9c	D;E	If predictor measurement requires subjective interpretation, describe the qualifications and demographic characteristics of the predictor assessors	-
Sample size	10	D;E	Explain how the study size was arrived at (separately for development and evaluation), and justify that the study size was sufficient to answer the research question. Include details of any sample size calculation	-
Missing data	11	D;E	Describe how missing data were handled. Provide reasons for omitting any data	7-8
Analytical methods	12a	D	Describe how the data were used (e.g., for development and evaluation of model performance) in the analysis, including whether the data were partitioned, considering any sample size requirements	7-8
	12b	D	Depending on the type of model, describe how predictors were handled in the analyses (functional form, rescaling, transformation, or any standardisation).	-
	12c	D	Specify the type of model, rationale[2], all model-building steps, including any hyperparameter tuning, and method for internal validation	7-8, 12
	12d	D;E	Describe if and how any heterogeneity in estimates of model parameter values and model performance was handled and quantified across clusters (e.g., hospitals, countries). See TRIPOD-Cluster for additional considerations[3]	-
	12e	D;E	Specify all measures and plots used (and their rationale) to evaluate model performance (e.g., discrimination, calibration, clinical utility) and, if relevant, to compare multiple models	7-9
	12f	E	Describe any model updating (e.g., recalibration) arising from the model evaluation, either overall or for particular sociodemographic groups or settings	-
	12g	E	For model evaluation, describe how the model predictions were calculated (e.g., formula, code, object, application programming interface)	-
Class imbalance	13	D;E	If class imbalance methods were used, state why and how this was done, and any subsequent methods to recalibrate the model or the model predictions	7-8,11-13
Fairness	14	D;E	Describe any approaches that were used to address model fairness and their rationale	-
Model output	15	D	Specify the output of the prediction model (e.g., probabilities, classification). Provide details and rationale for any classification and how the thresholds were identified	6-7

[1] D=items relevant only to the development of a prediction model; E=items relating solely to the evaluation of a prediction model; D;E=items applicable to both the development and evaluation of a prediction model

[2] Separately for all model building approaches.

[3] TRIPOD-Cluster is a checklist of reporting recommendations for studies developing or validating models that explicitly account for clustering or explore heterogeneity in model performance (eg, at different hospitals or centres). Debray et al, BMJ 2023; 380: e071018 [DOI: 10.1136/bmj-2022-071018]

Training versus evaluation	16	D;E	Identify any differences between the development and evaluation data in healthcare setting, eligibility criteria, outcome, and predictors	-
Ethical approval	17	D;E	Name the institutional research board or ethics committee that approved the study and describe the participant-informed consent or the ethics committee waiver of informed consent	-
**OPEN SCIENCE**				
Funding	18a	D;E	Give the source of funding and the role of the funders for the present study	1
Conflicts of interest	18b	D;E	Declare any conflicts of interest and financial disclosures for all authors	1-2
Protocol	18c	D;E	Indicate where the study protocol can be accessed or state that a protocol was not prepared	-
Registration	18d	D;E	Provide registration information for the study, including register name and registration number, or state that the study was not registered	-
Data sharing	18e	D;E	Provide details of the availability of the study data	16
Code sharing	18f	D;E	Provide details of the availability of the analytical code[4]	-
**PATIENT & PUBLIC INVOLVEMENT**				
Patient & Public Involvement	19	D;E	Provide details of any patient and public involvement during the design, conduct, reporting, interpretation, or dissemination of the study or state no involvement.	-
**RESULTS**				
Participants	20a	D;E	Describe the flow of participants through the study, including the number of participants with and without the outcome and, if applicable, a summary of the follow-up time. A diagram may be helpful.	-
	20b	D;E	Report the characteristics overall and, where applicable, for each data source or setting, including the key dates, key predictors (including demographics), treatments received, sample size, number of outcome events, follow-up time, and amount of missing data. A table may be helpful. Report any differences across key demographic groups.	8,9, 20; Suppl Table 1
	20c	E	For model evaluation, show a comparison with the development data of the distribution of important predictors (demographics, predictors, and outcome).	-
Model development	21	D;E	Specify the number of participants and outcome events in each analysis (e.g., for model development, hyperparameter tuning, model evaluation)	20; Suppl Table 1
Model specification	22	D	Provide details of the full prediction model (e.g., formula, code, object, application programming interface) to allow predictions in new individuals and to enable third-party evaluation and implementation, including any restrictions to access or re-use (e.g., freely available, proprietary)[5]	-
Model performance	23a	D;E	Report model performance estimates with confidence intervals, including for any key subgroups (e.g., sociodemographic). Consider plots to aid presentation.	9,10,11,12,22; SupplTables 3,5-8
	23b	D;E	If examined, report results of any heterogeneity in model performance across clusters. See TRIPOD Cluster for additional details[3].	-
Model updating	24	E	Report the results from any model updating, including the updated model and subsequent performance	-
**DISCUSSION**				
Interpretation	25	D;E	Give an overall interpretation of the main results, including issues of fairness in the context of the objectives and previous studies	14-16
Limitations	26	D;E	Discuss any limitations of the study (such as a non-representative sample, sample size, overfitting, missing data) and their effects on any biases, statistical uncertainty, and generalizability	15-16
Usability of the model in the context of current care	27a	D	Describe how poor quality or unavailable input data (e.g., predictor values) should be assessed and handled when implementing the prediction model	-
	27b	D	Specify whether users will be required to interact in the handling of the input data or use of the model, and what level of expertise is required of users	-
	27c	D;E	Discuss any next steps for future research, with a specific view to applicability and generalizability of the model	16

From: Collins GS, Moons KGM, Dhiman P, et al. *BMJ* 2024;385:e078378. doi:10.1136/bmj-2023-078378

[4] This relates to the analysis code, for example, any data cleaning, feature engineering, model building, evaluation.
[5] This relates to the code to implement the model to get estimates of risk for a new individual.
